# Supplementary material for: Epigenetic biomarkers to track differentiation of pluripotent stem cells
Source: Stem Cell Reports. 2022 Dec 1;18(1):145–58. doi: 10.1016/j.stemcr.2022.11.001 (PMC9860076; doi:10.1016/j.stemcr.2022.11.001)
Supplement: Document S1. Figures S1–S5 and Tables S4 and S5 [file mmc1.pdf]

Stem Cell Reports, Volume *18*

Supplemental Information

Epigenetic biomarkers to track differentiation of pluripotent stem cells

Marco Schmidt, Kira Zeevaert, Mohamed H. Elsafi Mabrouk, Roman Goetzke, and Wolfgang Wagner

Supplemental Figures

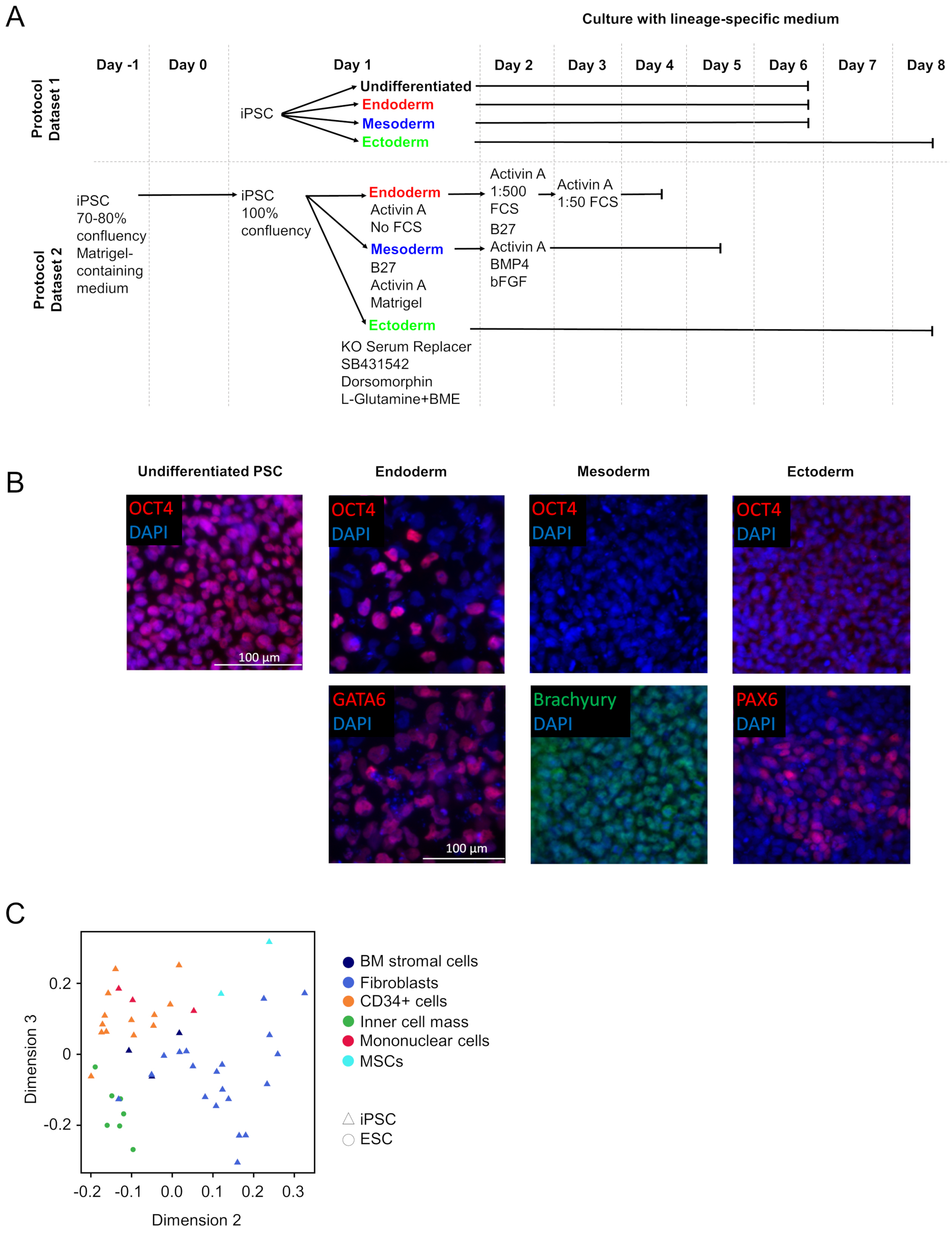

Figure S1: Trilineage differentiation of pluripotent stem cells (related to Figure 1)

(A) Trilineage differentiation protocols that were used for own iPSCs (dataset 1; STEMdiff Trilineage Differentiation Kit; Stemcell Technologies) and in the public dataset (dataset 2; GSE85828) (Daily et al., 2017).

(B) Immunofluorescence staining of exemplary differentiated cells (protocol dataset 1) stained with antibodies against OCT4 (stem cells), GATA6 (endoderm), Brachyury (mesoderm), and PAX6 (ectoderm). Nuclear staining with DAPI.

(C) Multidimensional scaling (MDS) plot of the top 10,000 most variable CpGs from own (dataset 1) and public DNAm profiles (dataset 2) (Daily et al., 2017) of undifferentiated stem cells only. Cell lines seem to cluster based on the cells used for reprogramming. SC12-040 was removed as being a prominent outlier.

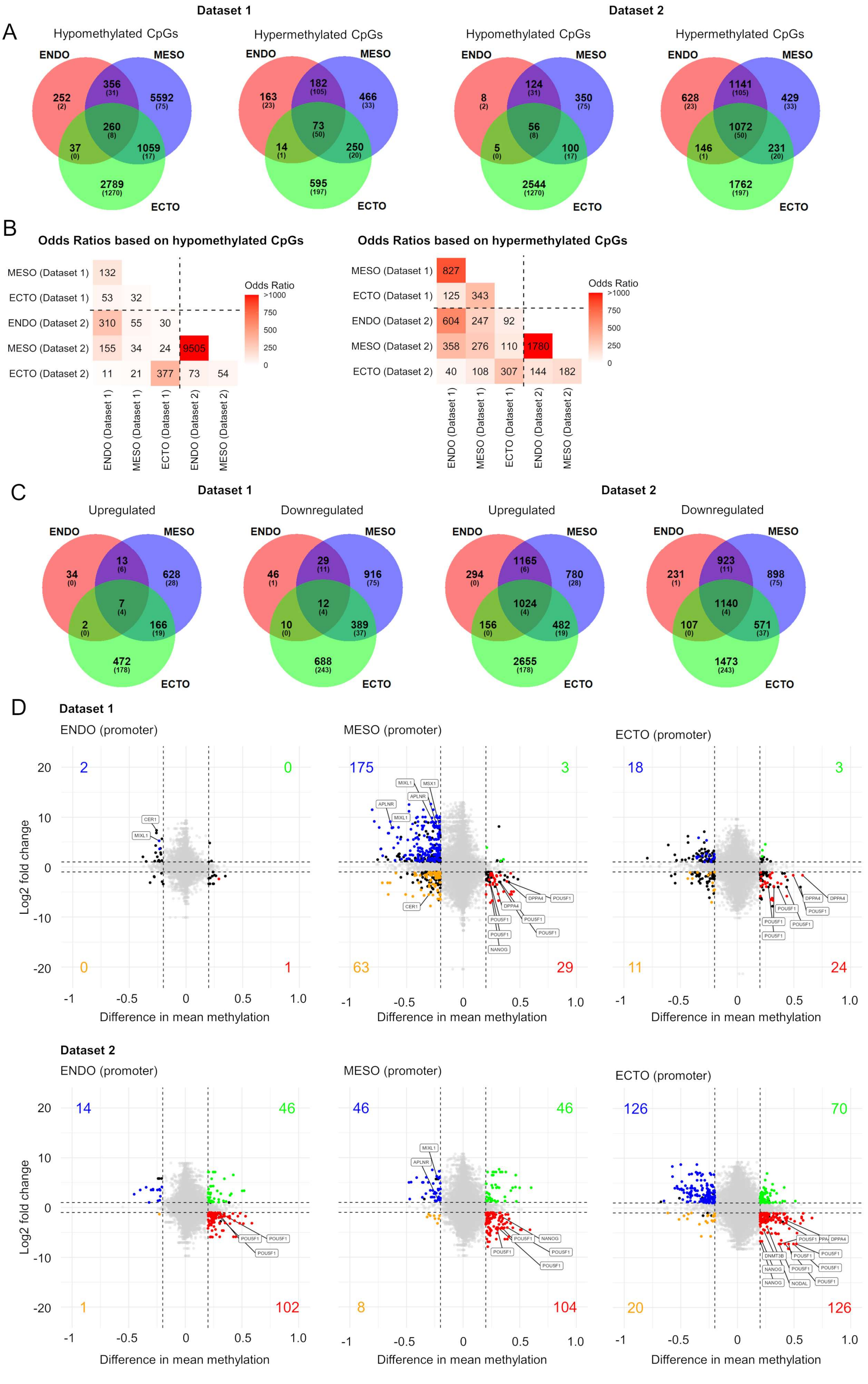

Figure S2 continues on the next page.

E

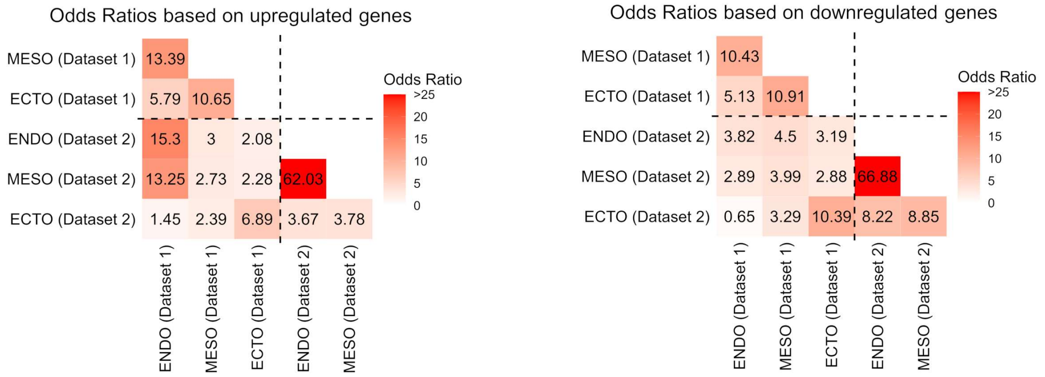

Figure S2: Overlap of DNA methylation changes in different datasets (related to Figure 1)

(A) Venn diagrams depict the number of significant hypo- and hypermethylated CpGs that are overlapping during differentiation towards endoderm (ENDO), mesoderm (MESO), and ectoderm (ECTO). This analysis was performed for dataset 1 and dataset 2, in parallel. Additionally, the overlap between the datasets is indicated in parenthesis.

(B) To estimate the relationship between the DNAm profiles of the differentiated samples we performed pairwise comparison of differentially methylated probes in datasets 1 and dataset 2 (for hypo- and hypermethylated sites, separately). The odds ratio of Fisher-Exact-Test provide the likelihood of getting the shared differentially methylated CpGs, given the total sample size (all CpGs). The high odds ratio between ENDO and MESO from dataset 2 indicates that the DNA methylation changes in these samples are highly overlapping.

(C) Venn diagrams provide the number of significant gene expression changes during differentiation into endoderm, mesoderm, and ectoderm. This analysis was performed for dataset 1 and dataset 2, separately. Additionally, the overlap between the datasets is indicated in parenthesis.

(D) Integrative analysis of DNA methylation and gene expression changes. Only CpGs in promoter regions (TSS1500, TSS200) are considered. Each dot represents a gene-CpG-pair (genes as well as CpGs might be duplicated). Colored dots depict pairs with a significant difference in DNAm and gene expression changes during differentiation.

(E) Heatmap of pair-wise odds ratio between differentially expression genes across differentiation modalities (Fisher-Exact Test). In dataset 2 the gene expression changes are significantly overlapping during endodermal and mesodermal differentiation.

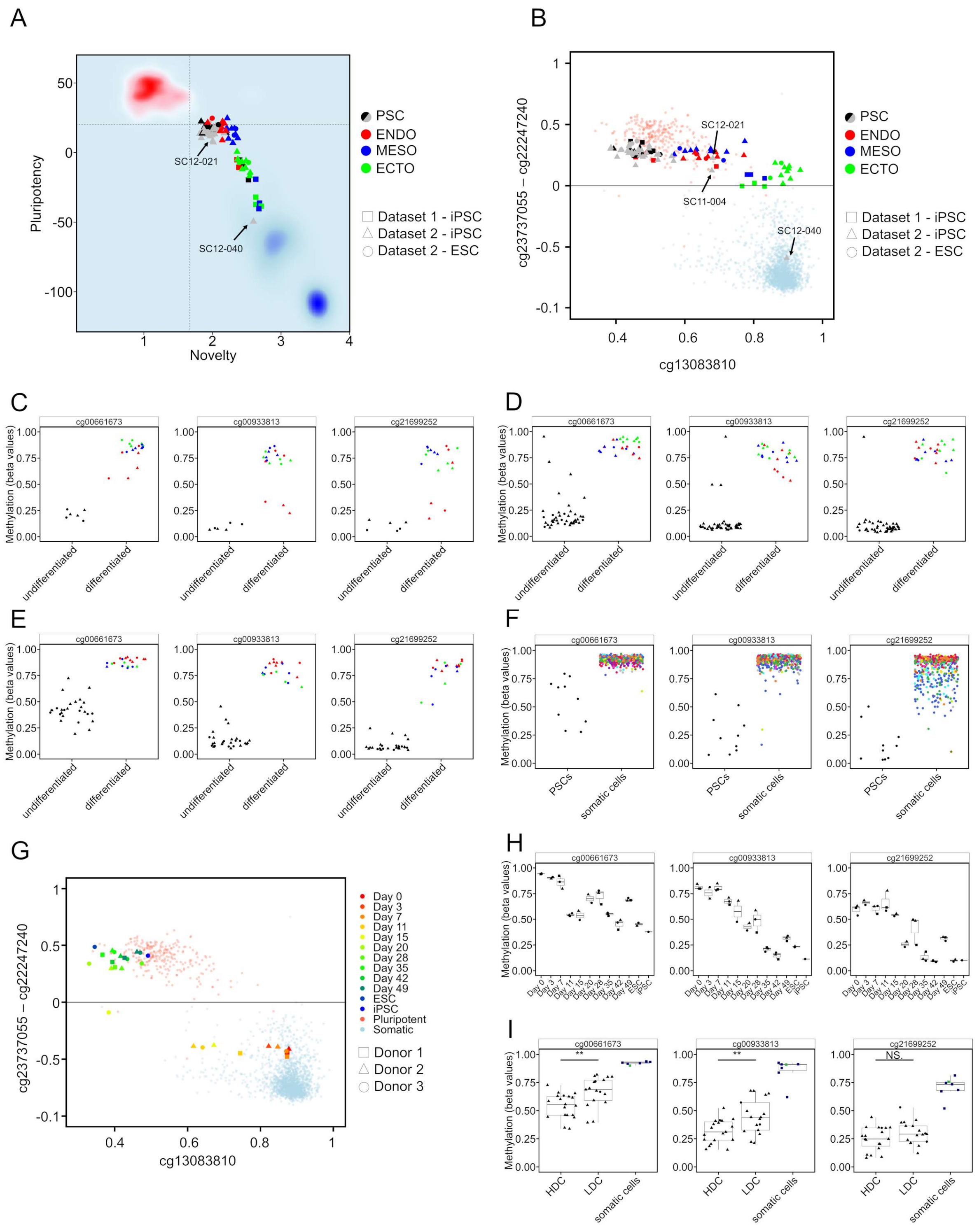

Figure S3, legend on the next page.

Figure S3: Comparison of different signatures for pluripotency (related to Figure 2)

(A) PluriTest analysis was performed with the online PluriTest tool (https://www.pluritest.org/). RNA-seq FASTAQ files were uploaded to the website for pre-processing, alignment, and automated analysis using the proprietary algorithm. The results showed that most stem cells, mesoderm, and endoderm samples had a similar pluripotency score. PluriTest was not able to distinguish between them and the predictions did not cluster with pluripotent cloud of the empirical density map. Furthermore, two of three iPSCs lines from dataset 1 and the outlier cell line SC12-040 had scores were indicated as failed.

(B) Epi-Pluri-Score analysis is based on DNAm at three specific CpGs. One of these CpGs was localized within the pluripotency-associated gene *POU5F1* (also known as *OCT4*). Furthermore, the difference in DNAm levels of CpGs in *ANKRD46* and *C14orf115* was determined and combined as Epi-Pluri-Score (Lenz et al., 2015). The dots in the background refer to DNAm profiles (all Illumina HumanMethylation27 BeadChip platform) of 264 pluripotent and 1,951 non-pluripotent cell preparations, respectively (Lenz et al., 2015). Notably, Epi-Pluri-Score classified all cell preparations as pluripotent, while early differentiation events can be tracked by increasing DNAm in *POU5F1.* For the three iPSC samples that were previously identified as outliers the sample IDs are again highlighted (GSM2285159, dataset 2). Particularly the sample SC12-040 was clearly classified as non-pluripotent, and this sample clustered also apart in the MDS and PCA plots (Figure 1A,C) and apparently also revealed an aberrant karyotype (Salomonis et al., 2016).

(C) DNA methylation levels for the undifferentiated and differentiated cells of the selection set for the three CpGs that were selected for the pluripotency score. Each of the CpGs could discern pluripotent and non-pluripotent cells.

(D) DNAm levels at the three candidate CpGs for the pluripotency score of the remaining samples from dataset 2 (Daily et al., 2017).

(E) DNAm levels at the three candidate CpGs for the pluripotency score in various iPSC-derived cell types (dataset 3; Table S1).

(F) DNAm levels at the three candidate CpGs for the pluripotency score for a collection of various somatic cell types (dataset 4; Table S1) (Schmidt et al., 2020).

(G) Epi-Pluri-Score analysis. Depicted are the same samples from Figure 2F and S3G. In contrast to the pluripotency score, the Epi-Pluri-Score changes abruptly between day 15 and 20.

(H) DNAm levels at the three candidate CpGs for the pluripotency score for a collection of samples during reprogramming of fibroblasts into iPSCs (dataset 5; Table S1) (Ohnuki et al., 2014).

(I) DNAm levels at the three candidate CpGs for the pluripotency score for iPSC samples (dataset 6; Table S1) (Butcher et al., 2016), which have been grouped into high differentiation capacity (HDC) and low differentiation capacity (LDC) toward endoderm. The primary donor samples (fibroblasts and endothelial precursors) are shown for comparison. P-values were calculated with Wilcoxon-Test (** $p < 0.01$).

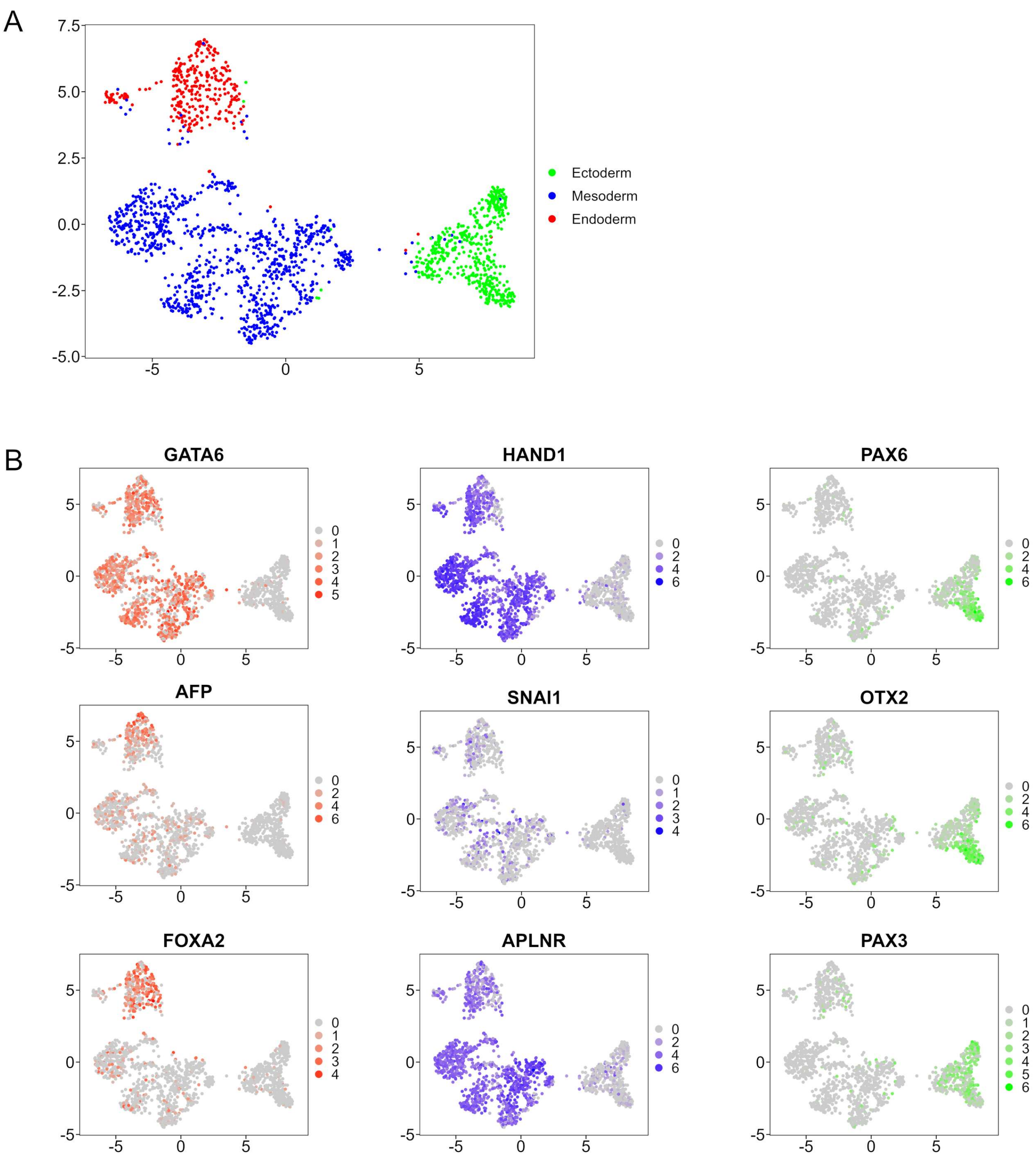

Figure S4: Identification of gene signatures for germ layers in EBs (related to Figure 4)

(A) To identify marker genes for endodermal, mesodermal, and ectodermal differentiation in spontaneous differentiation, we used single-cell RNA sequencing data of EBs at day eight of spontaneous differentiation (Han et al., 2018). Uniform Manifold Approximation and Projection (UMAP) representation of the data demonstrated that the cells could be classified in three distinct clusters.

(B) UMAP representation of normalized expression levels for endoderm markers (*GATA6*, *AFP*, *FOXA2*), mesoderm markers *(HAND1*, *SNAI1*, *APLNR*), and ectoderm markers (*PAX6*, *OTX2*, *PAX3*) demonstrates that these clusters were indeed associated with the respective germ layers. The same results were also observed for gene ontology analysis (not presented). For each of these clusters we subsequently selected genes that are significantly higher expressed than in the other clusters: for endoderm 279 genes, mesoderm 425 genes, and ectoderm 516 genes (Table S2).

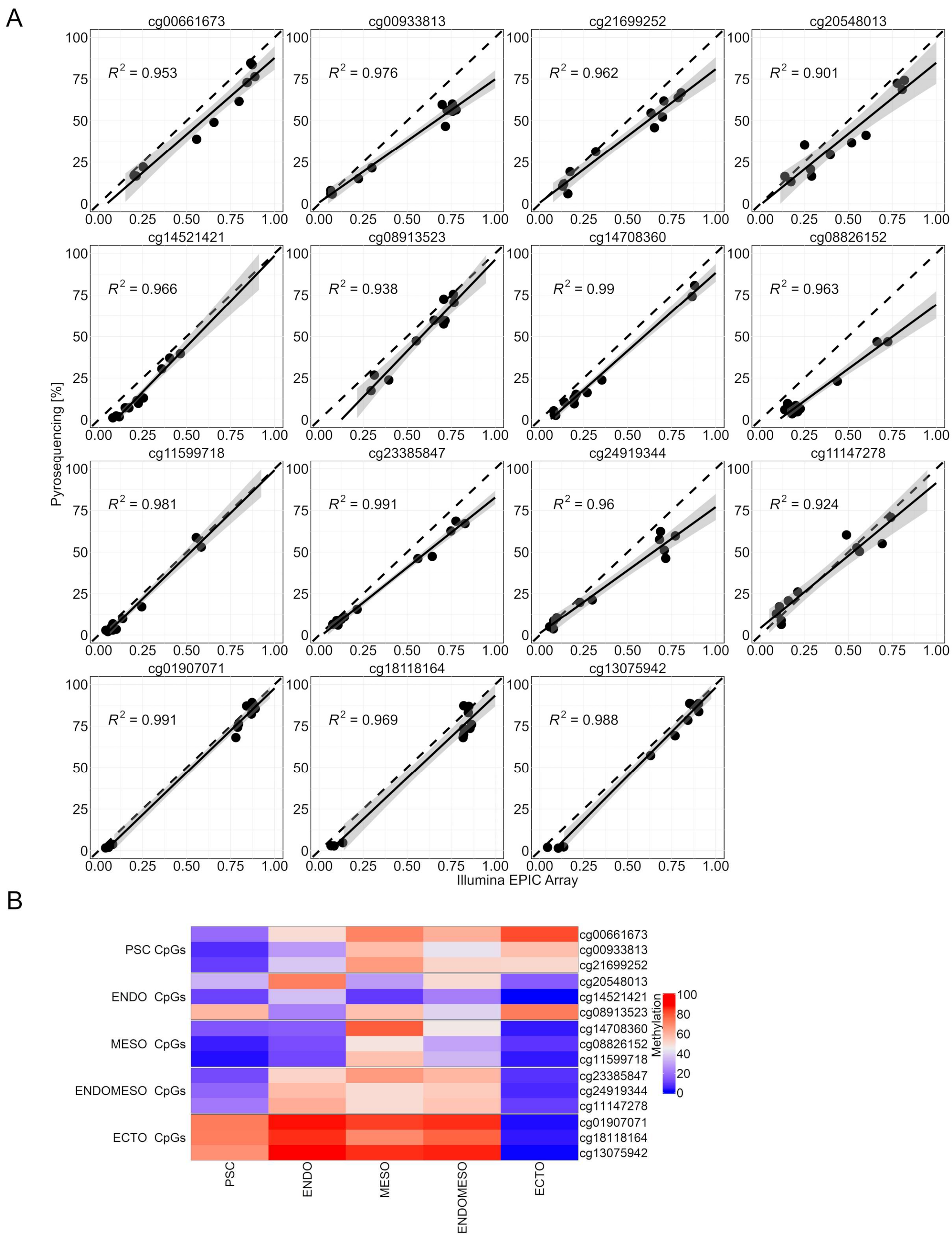

Figure S5: Pyrosequencing assays for GermLayerTracker CpGs (related to Figure 5)

(A) Comparison of DNAm values measured with pyrosequencing and the Illumina EPIC BeadChip technology.

(B) Reference values based on pyrosequencing for deconvolution of germ layers. Depicted are mean values of the three cell lines from the directed 2D differentiation.

Table S1: List of all datasets used for selection and validation of the DNA methylation signatures

This table is provided as separate Excel sheet.

Table S2: List of germ layer signature genes derived from publically available sc-RNA-seq data for embryoid bodies, related to Figure 4

This table is provided as separate Excel sheet.

Table S3: Excel sheet for deconvolution of germ layer composition

This table is provided as separate Excel sheet.

Table S4: Pyrosequencing primer, related to Figure 5.

NAME	DNA SEQUENCE
cgSC1 For	GGTTGGAGTGTATTGGTGTAA
cgSC1 Rev	Biotin-AATCCCAACCTTTATACATATTAATTCTT
cgSC1 Seq	GTTGAGATTATAGGTGTGA
cgSC2 For	AGGTTGGTTATGAATTTTTGGTTTTAAGTA
cgSC2 Rev	Biotin- ATACCCTACCTTCCTTTCATTTATATTC
cgSC2 Seq	TTGGGATTATAGGTGTG
cgSC3 For	GATGTTGAGGGTTAGGGGGTAATT
cgSC3 Rev	Biotin- CCTAAAACTCTAAAAATCTTTCTCCCTAAA
cgSC3 Seq	TGAAGGTTTTTTTAGTTTTGA
cgE1 For	GAATAGTATATGGTTGGTTGGGAAAGT
cgE1 Rev	Biotin- CCAAAAAAAAAAAAATACCTTTACTATCACT
cgE1 Seq	AGGAGTTATTTTATTATATTGGAG
cgE2 For	GGGATGTTGTGGATGGTAAAA
cgE2 Rev	Biotin- ACTCCCACATCTAAACACCTAA
cgE2 Seq	AGGGGTGTGGGAAGT
cgE3 For	GGGAGAGGGATTTATTATTAGGT
cgE3 Rev	Biotin- ACCCCCTCCTTCAACTATAAT
cgE3 Seq	GGTTTGAGAAAGAAGTTAG
cgM1 For	AGGGTAAGGTTGTTTTGTTTAGTTTAT
cgM1 Rev	Biotin- TCATACCTTTAAAACCCACAACTAAAAT
cgM1 Seq	ATTAGGGTTTTGGTTTTATT
cgM2 For	TGAGTTTGGTTAGTTTAGTTATAGGT
cgM2 Rev	Biotin- CATCCCTAAAACAAACAAAAAACAATT
cgM2 Seq	ATTTGTTGTTGAGGTTTTTAATA
cgM3 For	ATGGTTTGGTATAGAAAGTTTATGG
cgM3 Rev	Biotin- ATACTTTCATCTCTTCTAATACCTTTAAC
cgM3 Seq	GTTTTGTGGGTGGGG
cgEM1 For	GAATAAGATATGGTTTTTGGATTTGAGTA
cgEM1 Rev	Biotin- AAATTTTCCTCTCCTACATCTCTCA
cgEM1 Seq	GTGTTATAAGGTTTTGTTAGTT
cgEM2 For	Biotin- AGTTTTTTGATTATAAAAGGTATAGAGTGT
cgEM2 Rev	ACTCAAAAAAATCACCATAAATCACTATC
cgEM2 Seq	ACAACTAAACTTCTTTATCATATAT
cgEM3_C For	TGTTAGTAAATGGGGAAGATATAAAAGTT

cgEM3_C Rev	Biotin- AATTCCTACCCAACTCAAACTCATCTA
cgEM3_C Seq	GAGTTGATTTTGAAAGGT
cgEC1 For	GGGGTTTTGAAAGTAAATGTGT
cgEC1 Rev	Biotin- TTCCAACTCACTAAAAAACACTTC
cgEC1 Seq	AGTAAATGTGTTGAAAGTT
cgEC2 For	AGTGGGAGTAAATGAGTTTAGT
cgEC2 Rev	Biotin- CAATTTCAAAATCTCCATCTCAAAATATCA
cgEC2 Seq	TTTTAGGGTAAGAAAATATAGATAG
cgEC3 For	GGGAGATTTTAGTTTTTTTTGTAGGG
cgEC3 Rev	Biotin- CCCAATATTATAATTCTTAACACCTCTCAT
cgEC3 Seq	AGTTTTTTTTGTAGGGATTTT

Table S5: RT-qPCR primer, related to Figure 5.

NAME	DNA SEQUENCE
POU5F1 For	GGGGGTTCTATTTGGGAAGGTA
POU5F1 Rev	ACCCACTTCTGCAGCAAGGG
GATA6 For	CTCAGTTCCTACGCTTCGCAT
GATA6 Rev	GTCGAGGTCAGTGAACAGCA
TBXT For	CAGTGGCAGTCTCAGGTTAAGAAGGA
TBXT Rev	CGCTACTGCAGGTGTGAGCAA
PAX6 For	TCGAAGGGCCAAATGGAGAAGAGAAG
PAX6 Rev	GGTGGGTTGTGGAATTGGTTGGTAGA
GAPDH For	GAAGGTGAAGGTCGGAGTC
GAPDH Rev	GAAGATGGTGATGGGATTTC

Supplemental References

Daily, K., Ho Sui, S.J., Schriml, L.M., Dexheimer, P.J., Salomonis, N., Schroll, R., Bush, S., Keddache, M., Mayhew, C., Lotia, S., *et al.* (2017). Molecular, phenotypic, and sample-associated data to describe pluripotent stem cell lines and derivatives. Sci Data *4*, 170030.

Han, X., Chen, H., Huang, D., Chen, H., Fei, L., Cheng, C., Huang, H., Yuan, G.C., and Guo, G. (2018). Mapping human pluripotent stem cell differentiation pathways using high throughput single-cell RNA-sequencing. Genome Biol *19*, 47.

Lenz, M., Goetzke, R., Schenk, A., Schubert, C., Veeck, J., Hemeda, H., Koschmieder, S., Zenke, M., Schppert, A., and Wagner, W. (2015). Epigenetic biomarker to support classification into pluripotent and non-pluripotent cells. Scientific Reports *5*, 8973.

Ohnuki, M., Tanabe, K., Sutou, K., Teramoto, I., Sawamura, Y., Narita, M., Nakamura, M., Tokunaga, Y., Nakamura, M., Watanabe, A., *et al.* (2014). Dynamic regulation of human endogenous retroviruses mediates factor-induced reprogramming and differentiation potential. Proc Natl Acad Sci U S A *111*, 12426-12431.

Salomonis, N., Dexheimer, P.J., Omberg, L., Schroll, R., Bush, S., Huo, J., Schriml, L., Ho Sui, S., Keddache, M., Mayhew, C., *et al.* (2016). Integrated Genomic Analysis of Diverse Induced Pluripotent Stem Cells from the Progenitor Cell Biology Consortium. Stem Cell Reports *7*, 110-125.
