# Supplementary material for: Epigenetic biomarkers to track differentiation of pluripotent stem cells
Source: Stem Cell Reports. 2022 Dec 1;18(1):145–58. doi: 10.1016/j.stemcr.2022.11.001 (PMC9860076; doi:10.1016/j.stemcr.2022.11.001)
Supplement: Document S2. Article plus supplemental information [file mmc5.pdf]

Stem Cell Reports
Article
OPEN ACCESS

Epigenetic biomarkers to track differentiation of pluripotent stem cells

Marco Schmidt,[1,2,3] Kira Zeevaert,[1,2,3] Mohamed H. Elsafi Mabrouk,[1,2,3] Roman Goetzke,[1,2] and Wolfgang Wagner[1,2,*]
[1]Institute for Stem Cell Biology, RWTH Aachen University Medical School, 52074 Aachen, Germany
[2]Helmholtz-Institute for Biomedical Engineering, RWTH Aachen University Medical School, 52074 Aachen, Germany
[3]These authors contributed equally
*Correspondence: wwagner@ukaachen.de
https://doi.org/10.1016/j.stemcr.2022.11.001

SUMMARY

Quality control of induced pluripotent stem cells remains a challenge. For validation of the pluripotent state, it is crucial to determine trilineage differentiation potential toward endoderm, mesoderm, and ectoderm. Here, we report GermLayerTracker, a combination of site-specific DNA methylation (DNAm) assays that serve as biomarker for early germ layer specification. CG dinucleotides (CpGs) were identified with characteristic DNAm at pluripotent state and after differentiation into endoderm, mesoderm, and ectoderm. Based on this, a pluripotency score was derived that tracks reprogramming and may indicate differentiation capacity, as well as lineage-specific scores to monitor either directed differentiation or self-organized multilineage differentiation in embryoid bodies. Furthermore, we established pyrosequencing assays for fast and cost-effective analysis. In the future, the GermLayerTracker could be used for quality control of pluripotent cells and to estimate lineage-specific commitment during initial differentiation events.

INTRODUCTION

The hallmark of pluripotent stem cells (PSCs) is their ability to differentiate toward the three embryonic germ layers. To confirm pluripotency, the cells are usually either directed toward these lineages with specific culture media or with undirected multilineage differentiation assays in embryoid bodies (EBs) or teratomas (International Stem Cell Initiative, 2018). Marker genes can then be evaluated on gene expression or on protein level, such as *SOX17* and *GATA6* for endoderm, *GATA2* and *TBXT* (Brachyury) for mesoderm, or *SOX2* and *PAX6* for ectoderm (Gifford et al., 2013; O'Shea et al., 2020). The ScoreCard panel based on quantitative reverse transcription PCR (qRT-PCR) measurements of 96 genes can be used to determine early germ-layer-specific differentiation (Bock et al., 2011; Tsankov et al., 2015). Pluripotency can also be predicted based on gene expression profiles through PluriTest, a bioinformatic analysis of transcriptomes of undifferentiated cells (Muller et al., 2011). It reflects transcriptomic characteristics of PSCs, but it does not reveal differentiation capacity into specific germ layers (Bouma et al., 2017). A quantitative, robust, and scalable assay that can estimate germ layer-associated cell fractions in early differentiation is yet elusive. To this end, an epigenetic assay might be advantageous.

DNA methylation (DNAm) plays an important role in cellular differentiation and manifests during embryonic development (Bock et al., 2011). It occurs particularly at cytosine-guanine-dinucleotides (CpG sites), and these epigenetic modifications can be cell type specific (Roadmap Epigenomics Consortium et al., 2015). Since every cell has two DNA copies, DNAm is well suited to apply deconvolution algorithms to estimate the composition of different cell types (Wagner, 2022). This approach has been used for deconvolution of leukocyte subsets in blood (Frobel et al., 2018; Houseman et al., 2012; Sontag et al., 2022) or even of complex tissues (Moss et al., 2018; Schmidt et al., 2020). Furthermore, we have previously described the Epi-Pluri-Score, a signature that can discern pluripotent and non-pluripotent cells based on DNAm changes that occur at three specific CpG sites (Lenz et al., 2015). However, the Epi-Pluri-Score has not been designed to detect early differentiation events toward specific lineages.

In this study, we therefore aimed to identify characteristic DNAm signatures for each of the three germ layers. Notably, in some datasets transcriptomic and epigenetic changes during commitment toward mesoderm versus endoderm could hardly be discerned, which further substantiates the need for reliable biomarkers for specific cell fate decisions. Nevertheless, we could select three CpGs with characteristic DNAm for undifferentiated pluripotent cells, endoderm, mesoderm, ectoderm, and endomesoderm. Based on this, we developed GermLayerTracker: a tool consisting of a pluripotency score that may be indicative for differentiation potential and lineage-specific signatures to estimate fractions of early cell fate decisions in differentiation assays.

RESULTS

DNA methylation changes during directed germ layer specification

We differentiated three lines of induced pluripotent stem cells (iPSCs) toward endoderm, mesoderm, and ectoderm

This is an open access article under the CC BY license (http://creativecommons.org/licenses/by/4.0/).

to analyze their DNAm profiles with the Infinium EPIC BeadChips (Figures S1A and S1B; dataset 1). In addition, we utilized 114 DNAm profiles of iPSCs and embryonic stem cells (ESCs) that were differentiated with different protocols (dataset 2; 450K BeadChip platform; Table S1) (Daily et al., 2017). Multidimensional scaling (MDS) showed that iPSCs differentiated toward ectoderm clustered apart, whereas cells differentiated toward endoderm and mesoderm appeared most closely related (Figure 1A). Stem cells from dataset 1 appear to be clustering closer to the endodermal and mesodermal cells from dataset 2. However, it needs to be considered that this MDS analysis may also be affected by confounding effects, such as different cell types used for reprogramming, microarray platforms, and batch effects (Figure S1C).

Lineage-specific DNAm changes were initially analyzed for both datasets separately (for dataset 2, only PSCs with corresponding data for germ layer differentiation) (Daily et al., 2017). Many CpGs revealed significant hyper- or hypomethylation during differentiation toward endoderm, mesoderm, or ectoderm (Figure 1B, DNAm difference $\geq$20%, adjusted p values $\leq$ 0.05; Figure S2A). Notably, dataset 2 showed a very high overlap of DNAm changes toward mesoderm and endoderm (Figure S2B). Direct comparison of DNAm profiles from endoderm and mesoderm further substantiated that their epigenetic markup was very similar in dataset 2 (Figure 1C).

Corresponding RNA sequencing data revealed overall consistent gene expression changes during trilineage differentiation (Figure 1D). Many genes become significantly up- or downregulated during differentiation toward endoderm, mesoderm, and ectoderm (fold change >2; adjusted p value < 0.05; Figure S2C). In tendency, genes with hypomethylation in promoter regions showed upregulated gene expression, and vice versa (Figure S2D). In analogy to the DNAm data, there was a high overlap of differential gene expression during differentiation toward endoderm and mesoderm in dataset 2 (Figure S2E). Canonical markers for mesodermal differentiation were particularly upregulated in dataset 1 (Figure 1E). These results demonstrate that depending on the differentiation regimen, the differences between endoderm and mesoderm may only be marginal, which needs to be considered for identification of germ-layer-specific signatures.

Development of an epigenetic signature for pluripotent state

Established quality control measures for pluripotent cells should be able to detect early differentiation events. However, the previously described PluriTest (Muller et al., 2011) and Epi-Pluri-Score (Lenz et al., 2015) have been specifically developed to discern pluripotent and somatic cell types, while it remained unclear if these assays would also reliably capture the transcriptomic/epigenetic changes during early differentiation toward the three germ layers. We therefore applied PluriTest analysis to the RNA-seq profiles of datasets 1 and 2 (Figure S3A). Notably, PluriTest results of all samples—even the pluripotent iPSC and ESCs—did not cluster with the pluripotent samples of the reference cohort, which might be due to the fact that the assay was initially designed for a microarray platform that was meanwhile discontinued. Furthermore, endoderm and mesoderm differentiated cells had similar PluriTest results as the non-differentiated pluripotent cells. In analogy, Epi-Pluri-Score classified DNAm profiles of all samples as pluripotent, even those that were differentiated for few days toward endoderm, mesoderm, and ectoderm (Figure S3B). Taken together, PluriTest and Epi-Pluri-Score could not reliably capture early differentiation events.

For the development of GermLayerTracker, we therefore established a new pluripotency score based on the early DNAm changes during differentiation toward endoderm, mesoderm, and ectoderm. Relevant CpGs were selected for high difference in mean methylation and low variance within the groups with the R package CimpleG (Maié et al., 2022). As a selection set, we used the undifferentiated and differentiated samples of the three iPSC lines of our dataset 1 and three randomly selected PSC lines from dataset 2 to have balance between the studies. We decided to derive small epigenetic signatures, which facilitate targeted DNAm analysis, and therefore selected only three candidate CpGs for PSCs (Figure 2A). The three top CpGs were as follows: cg00661673, associated with the gene Palladin (*PALLD*); cg00933813, not associated with a specific gene; and cg21699252, associated with MYCN opposite strand (*MYCNOS*). The DNAm values at these sites were combined into a pluripotency score (sum of the DNAm levels and 1 − DNAm level for the hypomethylated sites), which could clearly separate PSC and the differentiated cells in the selection set (Figures 2B and S3C). Similar results were observed when we used the remaining samples of dataset 2 for initial validation (Figures 2C and S3D). Notably, the pluripotency score could also discern three samples that revealed deviations within the analysis of gene expression and DNAm profiles. In fact, extensive previous characterization of these cell lines revealed structural anomalies for SC12-040, decreased expression of pluripotency markers and spontaneous differentiation for SC12-021, and abnormal copy number variations that where not shared with the donor for SC11-004 (Salomonis et al., 2016). This exemplifies that our approach can support quality control. Furthermore, the pluripotency score could correctly separate PSCs and early differentiated cells in a completely independent collection of pluripotent and iPSC-derived cell types (dataset 3; Table S1 and Figure 2D). We also investigated somatic cells and found that the

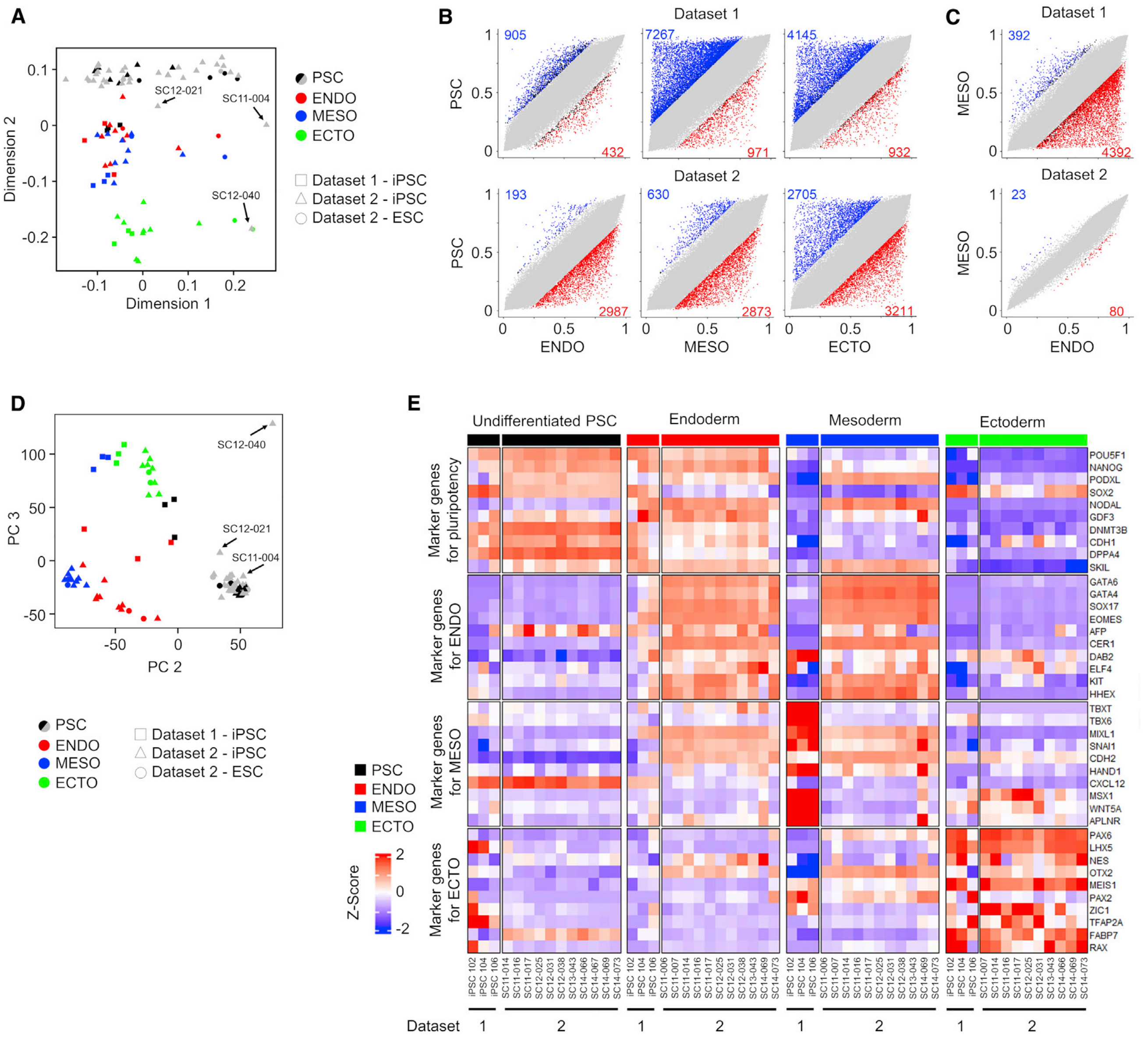

Figure 1. Germ-layer-specific DNA methylation during 2D iPSC differentiation

(A) Multidimensional scaling (MDS) plot of the top 10,000 most variable CpGs from own (dataset 1) and public DNAm profiles (dataset 2) (Daily et al., 2017). In gray are PSC lines from dataset 2 without corresponding differentiated samples. The names of three PSC outlier samples are depicted.

(B) Scatterplots of mean beta values (DNAm levels) at individual CpG sites for pairwise comparisons. Highlighted are CpGs ≥ 0.2 mean difference in beta values and colored if their adjusted p values are ≤ 0.05.

(C) Direct comparison of DNAm profiles from endoderm and mesoderm demonstrates that there is only a little difference between these differentiated cell types in dataset 2.

(D) Principal component analysis (PCA) of RNA-seq profiles of samples from our own (dataset 1) and public data (dataset 2) (Daily et al., 2017). The names of three PSC outlier samples are depicted.

(E) Gene expression of canonical germ layer marker genes (ten for each lineage) in samples from our own (dataset 1) and public datasets (dataset 2). The heatmap depicts *Z* score of vst-transformed read counts. See also Figures S1 and S2.

pluripotency score was consistently very low in primary cell types (549 DNAm profiles compiled from 21 studies; Schmidt et al., 2020; dataset 4; Figures 2E and S3F).

Next, we investigated how the pluripotency score changes in the course of reprogramming. To this end, we analyzed a dataset of three fibroblast lines, which were

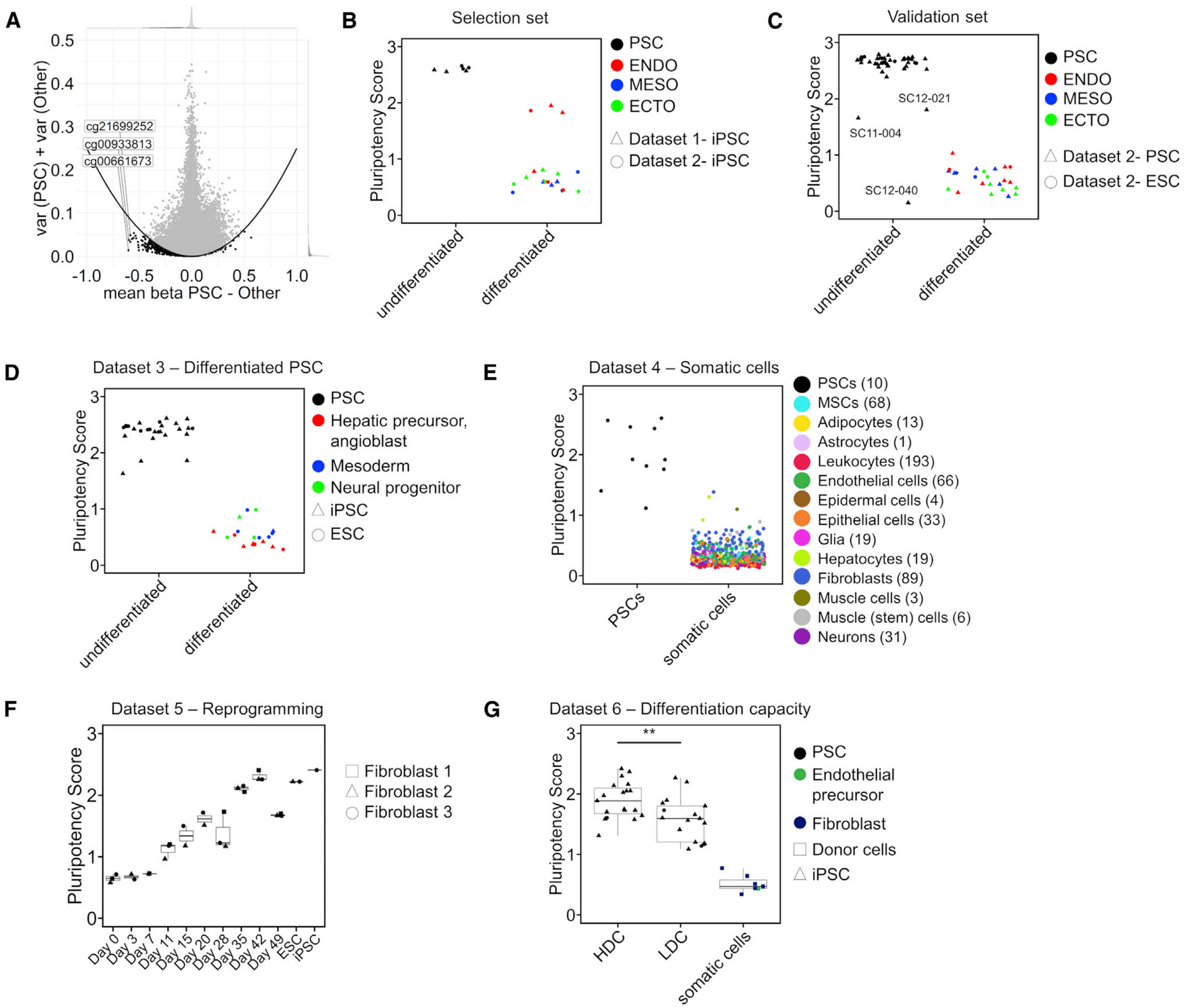

Figure 2. Derivation of a new pluripotency score based on DNAm at three CpGs

(A) The DNAm profiles of pluripotent stem cells (PSC) were compared with all other differentiated cell types (endoderm, mesoderm, and ectoderm) to identify three candidate CpGs. The difference in mean beta values (DNAm levels) is plotted against the combined variance within the groups. The parabola is part of the selection process (mean parabola parameter exemplarily visualized).

(B) The pluripotency score of the samples used for CpG selection. The score is a sum of 1 – DNAm of the individual specifically hypomethylated CpGs.

(C) The pluripotency score of the remaining samples from dataset 2 (Daily et al., 2017).

(D) The pluripotency score of various iPSC-derived cell types (dataset 3; Table S1).

(E) The pluripotency score for a collection of various somatic cell types (dataset 4; Table S1) (Schmidt et al., 2020).

(F) The pluripotency score for three different fibroblast lines during reprogramming into iPSCs (GEO: GSE54848; dataset 5) (Ohnuki et al., 2014).

(G) The pluripotency score for iPSC samples (GEO: GSE59091, dataset 6) (Butcher et al., 2016), which have been grouped into high differentiation capacity (HDC) and low differentiation capacity (LDC) toward endoderm. The primary donor samples (fibroblasts and endothelial precursors) are shown for comparison. p values were calculated with Wilcoxon test (**$p < 0.01$). Values depicted are means of multiple replicates. See also Figure S3.

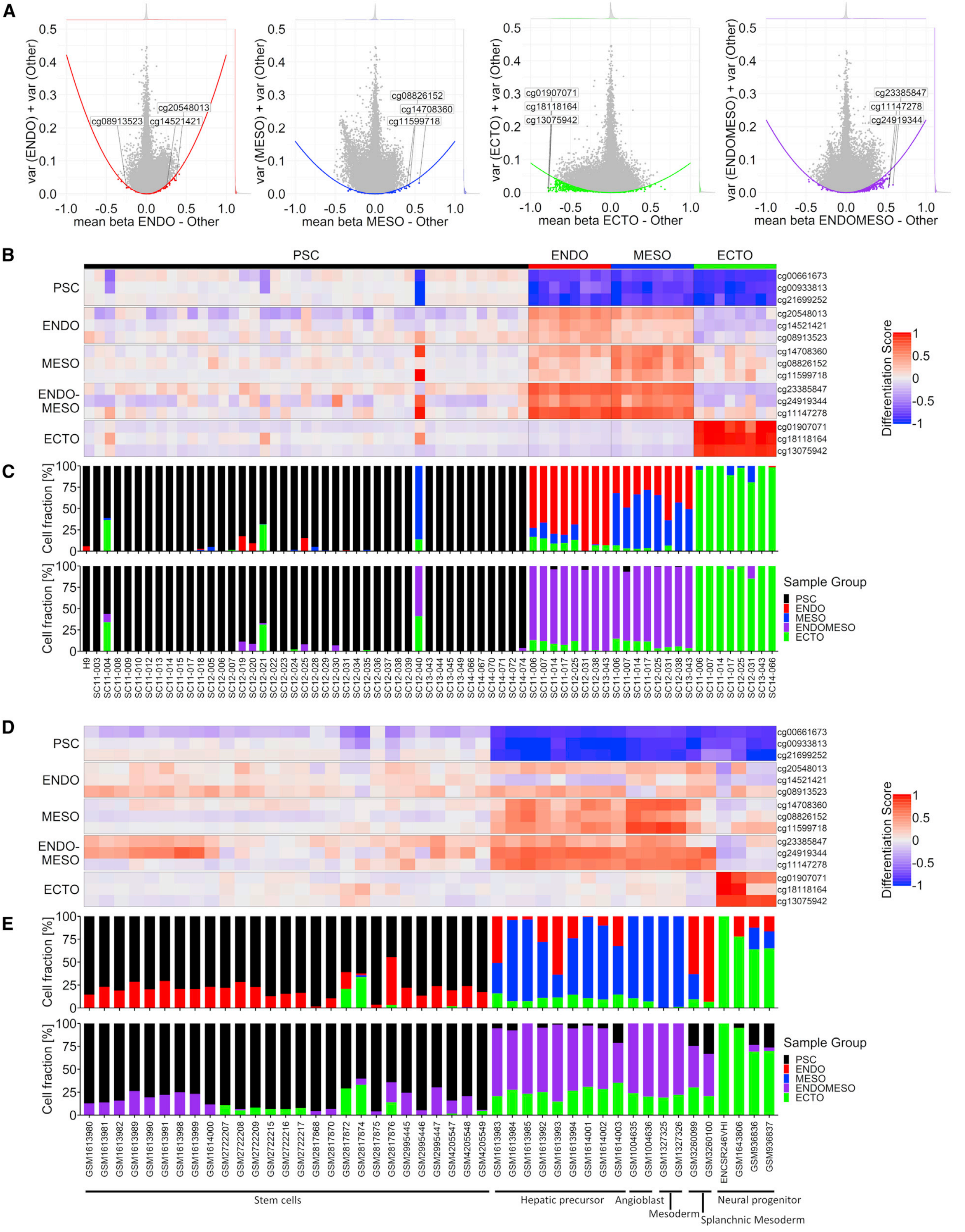

(legend on next page)

reprogrammed with retroviral vectors and sorted for TRA-1-60 expression at various time points (dataset 5) (Ohnuki et al., 2014). We have previously demonstrated that most pluripotency-associated genes in this dataset reveal consistent DNAm changes between day 15 and day 20 (Franzen et al., 2021), which is also exemplified by a drastic change in Epi-Pluri-Score analysis (Figure S3G). In contrast, the pluripotency score increases linearly during reprogramming until reaching similar levels compared with ESCs and iPSCs at day 42 (Figures 2F and S3H). This indicates that our selected sites are a good indicator for the reprogramming status of these cells.

Finally, we benchmarked our pluripotency score on DNAm profiles of PSCs that fulfilled criteria for pluripotency but revealed either higher differentiation capacity (HDC) or lower differentiation capacity toward endoderm (LDC; GEO: GSE59091, dataset 6) (Butcher et al., 2016). Notably, the pluripotency score was overall significantly higher in HDC than LDC pluripotent cells (p value = 0.009; Figures 2G and S3I). These findings indicate that the pluripotency score not only discerns early differentiation steps but might also provide a quality measure for pluripotent differentiation potential. This still needs to be further validated with more cell lines and with regard to three-lineage differentiation potential.

Selection of germ-layer-specific CpG sites

Since our biomarker should also reflect the specific differentiation toward endoderm, mesoderm, or ectoderm, we selected candidate CpGs for each germ layer as well. To this end, we used the same selection set as for the pluripotency score and applied the same selection method for each differentiated cell type (Figure 3A). Based on this, we selected the three top CpGs for endoderm (ENDO): cg20548013, associated with phosphatase and actin regulator 1 (*PHACTR1)*; cg14521421, associated with DENN domain containing 2B *(DENND2B);* and cg08913523 (no gene); for mesoderm (MESO) the CpGs cg14708360 (no gene); cg08826152, associated with adenosine receptor A2B (*ADORA2B);* and cg11599718 associated with vacuolar protein sorting-associated protein 37B (*VPS37B)*; for ectoderm (ECTO) the CpGs cg01907071, associated with the gene thrombospondin type 1 domain containing 4 (*THSD4)*; cg18118164, associated with ephrin A5 (*EFNA5)*; and cg13075942, associated with RAD51 Paralog B (*RAD51B)*. Since endoderm and mesoderm were closely related, particularly in dataset 2, we also selected candidate CpGs for a combination of endoderm and mesoderm (ENDOMESO): cg23385847, associated with the gene calcium/calmodulin dependent protein kinase IV (*CAMK4*); cg24919344 (no gene); and cg11147278 (no gene).

These candidate CpGs were subsequently validated in the remaining samples of dataset 2. Since the CpGs reveal cell-type-specific hypo- or hypermethylation, we used the complementary DNAm levels (1 – DNAm) for hypomethylated sites to provide differentiation scores that increase with differentiation. These scores were calculated as difference to the mean DNAm level of undifferentiated samples (Figure 3B). Alternatively, we estimated the fraction of lineage-specific differentiation in the cell population by deconvolution with a non-negative least squares approach, which is however hampered by the fact that we are looking for early germ layer specification and not for a defined endpoint in differentiation and by the discrepancy of the outcome between different germ layer differentiation protocols. Either way, the deconvolution approach classified most samples correctly into the categories PSC, ENDO, MESO, ECTO, and ENDOMESO (Figure 3C). The three outlier PSC samples could again be discriminated by higher differentiation scores. Furthermore, the individual DNAm levels as well as the deconvolution approach classified most iPSC-derived cells correctly that have been differentiated toward various cell types (dataset 3; Figures 3D and 3E).

DNA methylation changes in embryoid bodies

To determine if GermLayerTracker would also capture the germ-layer-specific epigenetic modifications during spontaneous differentiation, we generated EBs and analyzed DNAm profiles before (day 0), at day 4, and at day 7 after

Figure 3. Selection of germ-layer-specific CpG sites

(A) Selection of candidate CpGs for endoderm (ENDO), mesoderm (MESO), ectoderm (ECTO), and endomesoderm (ENDOMESO) in the selection set. The difference in mean beta values (DNAm levels) is plotted against the combined variance within the groups. The mean parabola parameter is exemplarily visualized and the selected candidate CpGs indicated.

(B) The heatmap depicts differentiation scores of the CpG sites for the remaining samples from dataset 2. The scores show the differences of the DNAm levels to the reference stem cells (for hypomethylated CpGs, 1 – DNAm was calculated); white means no change in comparison to stem cells, red means a change toward specific methylation and blue vice versa.

(C) Deconvolution results (with either ENDO and MESO in upper row, or the ENDOMESO in lower row) based on a non-negative least squares approach.

(D) Differentiation scores for iPSC-derived cells that were differentiated toward different cell types (dataset 3; Table S1; formatted in analogy to B).

(E) Deconvolution results for dataset 3 (formatted in analogy to C).

aggregation (EB dataset 1; Table S1). As expected, the three CpGs that are specific for the undifferentiated state change already within 4 days of undirected EB differentiation, which is reflected by a rapid decline of the pluripotency score (Figure 4A), indicating that most of the cells did exit the pluripotent state. Furthermore, the differentiation scores (Figure 4B) pointed toward differentiation in the three germ layers. However, the ENDOMESO-associated CpG site cg2338547 did not reveal the expected hypermethylation in this dataset, resulting in a discrepancy in deconvolution predictions (Figure 4C).

Furthermore, we used public DNAm profiles of EBs at day 17 (EB dataset 2; Table S1) (Daily et al., 2017). The differentiation scores and deconvolution results again demonstrated gain of lineage-specific epigenetic patterns. The strong changes in the MESO CpGs dominated deconvolution results. Notably, the results indicated that some of the EBs differentiated more toward ectodermal or endodermal lineage (Figure 4D). This finding may reflect the different propensity of each iPSC line to differentiate preferentially toward one or the other cell line.

We then used the available gene expression data from the day 17 EBs (Daily et al., 2017) to determine if lineage-specific bias is also reflected in the transcriptome. To this end, we have first identified gene signatures that are characteristic for the different germ layers during spontaneous differentiation. Public single-cell RNA sequencing data derived from EBs at day 8 clustered according to the germ layers (Figure S4) (Han et al., 2018). Based on this, we selected gene lists that are most prominently associated with the endoderm, mesoderm, and ectodermal cluster (Table S2). In fact, the genes of this ectodermal signature were overall higher expressed in EBs that were also predicted to have ectodermal bias in the GermLayerTracker (Figure 4E).

Targeted assays with pyrosequencing

Subsequently, we designed pyrosequencing assays for targeted analysis of the relevant CpGs to make GermLayerTracker applicable without the need of Illumina BeadChip analysis. When we reanalyzed the samples from the directed differentiation, the DNAm levels showed little deviation between pyrosequencing and EPIC BeadChip measurements (Figure S5A). Either way, we used the pyrosequencing results to adjust the reference matrix for deconvolution (Figure S5B). To further benchmark this assay, we again generated EBs and cultured them for 5 or 15 days. We also used our iPSC lines with PRDM8 knockout (*PRDM8*$^{-/-}$) that have been shown to reveal lower propensity to differentiate toward neurons (Cypris et al., 2020). Furthermore, we used in-house iPSC lines with YAP1 knockout (*YAP*$^{-/-}$) that hardly differentiated toward ectoderm (Zeevaert et al., 2022), and this phenotype has recently also been described by others (Stronati et al., 2022). The pyrosequencing measurements for GermLayerTracker could clearly discern non-differentiated pluripotent cells from either directed differentiation or EBs (Figure 5A). Furthermore, the differentiation scores clearly demonstrated that EBs of *PRDM8*$^{-/-}$ and *YAP*$^{-/-}$ did not acquire the typical ectoderm-associated DNAm. The deconvolution results for these knockout lines demonstrated lower fractions of ectoderm, accordingly (Figure 5B). To further benchmark our results, we have also analyzed gene expression of germ-layer-associated genes in these samples with qRT-PCR: *POU5F1* (OCT4) for pluripotent cells, *GATA6* for endoderm, *TBXT* (Brachyury) for mesoderm, and *PAX6* for ectoderm (Figure 5C). Furthermore, we performed ScoreCard assays for selected samples (Figure 5D). Overall, the predictions with GermLayerTracker were in line with the results from qRT-PCR and the ScoreCard for the knockout cell lines.

DISCUSSION

Quality measures of iPSC lines can be used for different objectives: (1) to monitor initial reprogramming of somatic cells, (2) to determine differentiation capacity of non-differentiated cells, and (3) to track differentiation to ultimately validate pluripotent differentiation potential (Steeg et al., 2021).

Initial monitoring of reprogramming often relies on microscopic assessment of colony morphology or upregulation of individual markers by immunofluorescence or qRT-PCR, but these approaches are difficult to quantify and lack standardized thresholds. A broader gene expression signature, such as PluriTest (Muller et al., 2011), can provide a more robust measure for successful reprogramming. However, when we applied the online PluriTest tool using the proprietary algorithm for pre-processing of RNA-seq results, the non-differentiated iPSCs were not clearly associated with the highlighted area for pluripotency in the empirical density map, and early differentiation events were not reliably detected. Our previously described Epi-Pluri-Score can provide a good alternative to validate reprogramming into pluripotent state (Lenz et al., 2015). In fact, it could clearly discern the three iPSC lines SC12-040, SC12-021, and SC11-004, which apparently did not resemble normal pluripotent cell lines (Salomonis et al., 2016). However, there was so far no epigenetic biomarker to detect early germ-layer-specific cell fate decisions.

In this study, we describe GermLayerTracker, which was—in contrast to the above-mentioned approaches—specifically designed to detect early differentiation events. Although the signature was not developed on somatic cells,

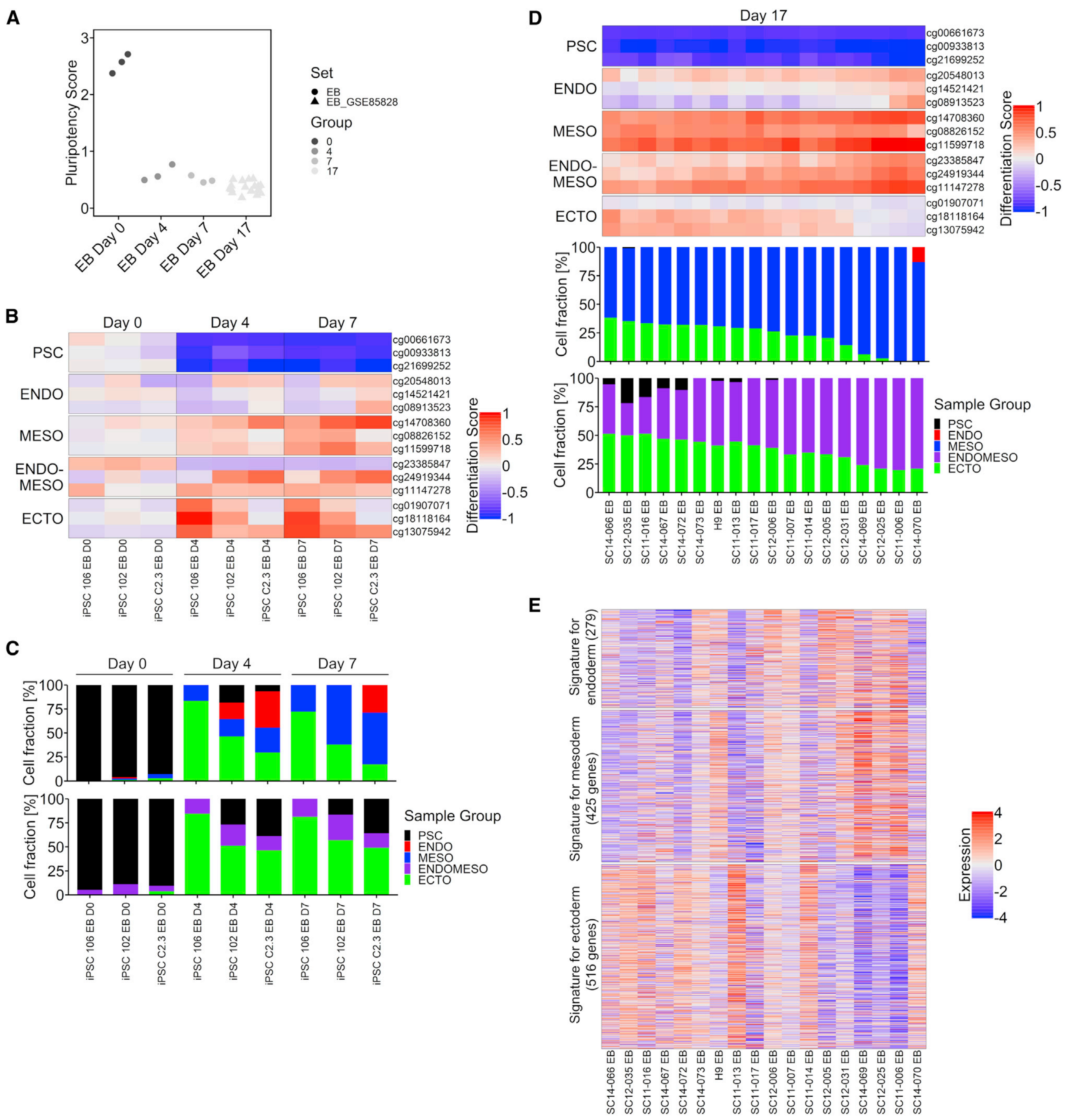

Figure 4. Pluripotency and differentiation scores in embryoid bodies

(A) Pluripotency score of the EBs from before (day 0), after 4 days, after 7 days, and after 17 days of differentiation.

(B) Differentiation scores for the same samples demonstrate that most, but not all CpGs, reveal lineage-specific DNAm changes. The scores show the differences of the DNAm levels to the reference stem cells (for hypomethylated CpGs, 1 − DNAm was calculated).

(C) Deconvolution results with either ENDO and MESO CpGs (in upper row) or the ENDOMESO CpGs (in lower row).

(D) Differentiation scores and deconvolution results for EBs after 17 days of culture (Daily et al., 2017). The samples are ordered based on the fraction of ectoderm in the deconvolution results.

(E) Heatmap depicts *Z* scores gene expression signatures for endoderm (279 genes), mesoderm (425 genes), and ectoderm (516 genes) in the corresponding EBs. These signatures were derived from a public single-cell RNA sequencing dataset for D8 EBs (Han et al., 2018). Overall, EBs with ectodermal bias in our epigenetic scores revealed also higher expression of ectodermal gene expression. See also Figure S4.

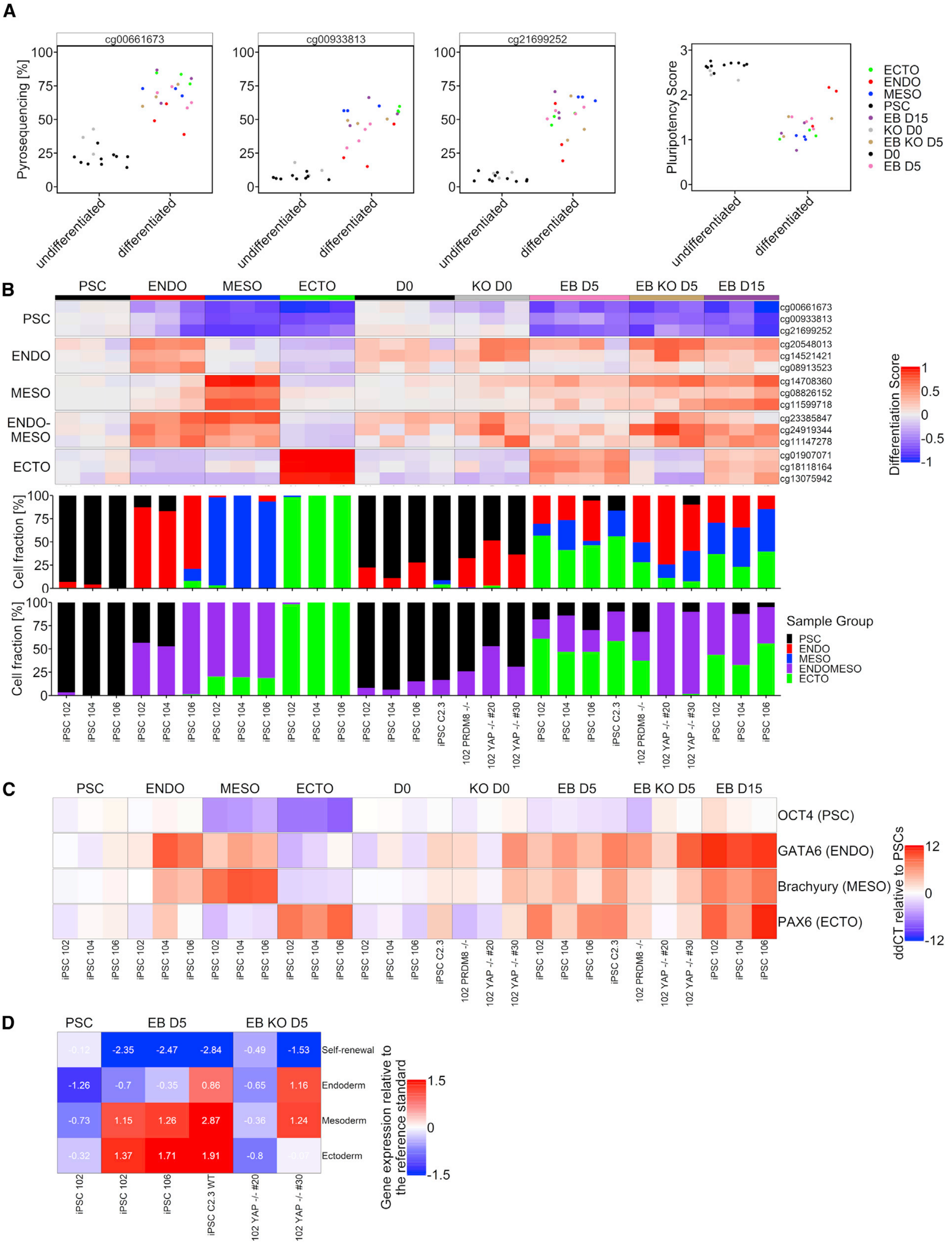

(legend on next page)

it could reliably discern somatic and pluripotent cells. In contrast to our previous Epi-Pluri-Test, the pluripotency score gradually increased during reprogramming until day 42 and might therefore better monitor the state of reprogramming. Furthermore, the pluripotency score was overall higher in iPSCs with HDC versus LDC toward endoderm (Butcher et al., 2016), which might suggest that the method could also be applied to estimate the differentiation capacity of the non-differentiated iPSCs. However, in this dataset the differentiation capacity toward mesoderm and ectoderm has not been addressed. It will therefore be necessary to further validate if the GermLayerTracker might be indicative for the differentiation potential of iPSCs already under pluripotent culture conditions. To this end, it would also be necessary to better define thresholds.

So far, validation of trilineage differentiation potential requires upfront differentiation with either directed or spontaneous differentiation for subsequent analysis. The teratoma assay is a method to test pluripotency by transplanting PSCs into an immunodeficient mouse where they will spontaneously form germ cell tumors with immunophenotypic characteristics of all germ layers (International Stem Cell Initiative, 2018). This assay raises concerns for animal welfare, analysis takes several months, and it is costly. Moreover, teratoma formation has high variability and can hardly be quantified (Dolgin, 2010; Muller et al., 2010; Tsankov et al., 2015). The ScoreCard assay is based on directed or spontaneous differentiation regimen and utilizes a relatively large panel of references genes. In analogy, our GermLayerTracker could track early lineage decisions in directed differentiation. Furthermore, it could be used to estimate the cellular composition in EBs. The predicted ectodermal fractions correlated in gene expression profiles and deconvolution results. Furthermore, iPSC lines with impaired undirected ectodermal differentiation, such as *PRDM8*$^{-/-}$ and *YAP*$^{-/-}$ lines could be identified.

Many laboratories are more used to gene expression analysis compared with DNAm analysis. However, targeted DNAm analysis is also feasible with other methods, such as EpiTYPER, digital droplet PCR, or amplicon deep sequencing (Han et al., 2020). To this end, a method could also be established for these alternative instruments in the future. Handling and shipment of DNA samples is easier than that of RNA samples, which would be a benefit for centralized analysis, e.g., by a service provider. GermLayerTracker is only based on 12 CpGs (with ENDOMESO 15 CpGs). Such small signatures are a trade-off since they may be more susceptible to individual outliers than signatures that integrate hundreds of CpGs. On the other hand, such targeted assays can be measured in a cost-effective and robust manner, independent of specific microarray platforms or bioinformatic tools. This is important if such assays should be utilized for clinical validation of therapeutic cellular products, which may even require accreditation as an *in vitro* diagnostic device (Wagner, 2022). In fact, we and others have demonstrated before that even individual CpGs may provide reliable biomarkers (Schmidt et al., 2020; Sontag et al., 2022), and it is therefore conceivable to further narrow down the CpGs of GermLayerTracker.

We were facing various challenges during this study. (1) The number of available datasets was limited, and (2) it was unexpected that the directed differentiation regimen with different protocols resulted in quite different DNAm and gene expression profiles; it even was difficult to reliably discern endoderm and mesoderm in dataset 2. In the future, additional datasets should be generated with alternative differentiation regimen to better identify and validate specific DNAm changes for endoderm and mesoderm. (3) The DNAm changes during directed differentiation with differentiation media do not necessarily reflect spontaneous differentiation in EBs. Sorting of cells would be advantageous to further adjust the signatures for spontaneous differentiation. (4) For validation of the deconvolution approach, there is no dataset available with quantitative data for lineage commitment in EBs. Specific DNAm patterns have been successfully used for deconvolution of cell populations, e.g., for the composition of leukocyte subsets (Frobel et al., 2018; Houseman et al., 2012;

Figure 5. Targeted assays of selected CpGs measured with pyrosequencing

(A) Pluripotency scores of samples measured with pyrosequencing. The methylation values of the three CpGs are also shown individually. Here, we used the same iPSCs as for the BeadChips with directed differentiation toward endoderm (ENDO), mesoderm (MESO), and ectoderm (ECTO), as well as EBs before, after 5 days, and after 15 days of spontaneous differentiation in suspension culture (D0, D5, and D15, respectively). In addition, three knockout (KO) iPSC lines were used (two *YAP*$^{-/-}$ and *PRDM8*$^{-/-}$).

(B) Differentiation scores for these samples was measured with pyrosequencing. The scores show the differences of the DNAm levels to the reference stem cells (for hypomethylated CpGs, 1 – DNAm was calculated). The deconvolution results with either ENDO and MESO or the ENDOMESO CpGs are also shown.

(C) qRT-PCR results of germ-layer-specific genes. The heatmap depicts ddCT values compared with the stem cells. Values depicted are means of technical replicates.

(D) ScoreCard results. Shown are the combined gene expression scores for each germ layer relative to the reference standard for selected iPSC lines. See also Figure S5.

Sontag et al., 2022), or even of complex tissues (Moss et al., 2018; Schmidt et al., 2020), but all of these applications were applied on terminally differentiated cells. In contrast, the differentiation process of iPSCs rather resembles a continuum without a fixed endpoint when the early germ layer differentiation is complete. The deconvolution results of GermLayerTracker can therefore not reflect the absolute composition of different cell types, but rather they provide a surrogate marker to estimate early cell fate decisions.

Taken together, our analysis provides further insight into epigenetic changes in early cell fate decisions. We established candidate CpGs for assessment of PSC at pluripotent state and to capture early cell fate decisions toward endoderm, mesoderm, and ectoderm. GermLayerTracker provides various advantages when compared with conventional methods for quality control of iPSCs. Such analysis can also be used for optimization of culture conditions to maintain a larger proportion of cells in pluripotent state or to better direct differentiation toward specific germ layers.

EXPERIMENTAL PROCEDURES

Resource availability

Corresponding author

Further information and requests for resources and reagents should be directed to and will be fulfilled by the corresponding author, Wolfgang Wagner (wwagner@ukaachen.de).

Materials availability

This study did not generate new unique reagents.

Data and code availability

The generated RNA-seq and methylation data are available on the Gene Expression Omnibus (https://www.ncbi.nlm.nih.gov/geo/) under the accession number GSE207119. The current study did not generate any original code. Additional information required to reanalyze the data can be provided upon request from the corresponding author.

Cell culture and directed differentiation

Four human iPSC lines were generated by reprogramming with episomal plasmids from bone-marrow-derived mesenchymal stromal cells (iPSC 102, iPSC 104, iPSC 106 (Goetzke et al., 2018), which can be found on hPSCreg under UKAi009-A, UKAi010-A, and UKAi011-A) or dermal fibroblast (TF11-C2.3) (Willmann et al., 2013). All samples were taken after informed and written consent using guidelines approved by the Ethic Committee for the Use of Human Subjects at the University of Aachen (permit number: EK128/09). The iPSC lines were cultured on tissue culture plastic coated with vitronectin (0.5 $\mu g/cm^2$) in StemMACS iPS-Brew XF (Miltenyi Biotec, Bergisch Gladbach, Germany). Directed differentiation toward endodermal, mesodermal, and ectodermal lineage was induced with the STEMdiff Trilineage Differentiation Kit (Stemcell Technologies, Vancouver, Canada; Figure S1A).

Embryoid body formation

Self-detaching iPSCs were generated, as described before (Elsafi Mabrouk et al., 2022). In brief, vitronectin was micro-contact printed (diameter 600 μm), and iPSCs grew and self-organized on these substrates. After about 6 days, when more than 50% of the colonies detached, the floating aggregates were harvested and considered as day 0 for further differentiation steps. Alternatively, Spin-EBs were generated as described previously (Ng et al., 2005). Non-directed multilineage differentiation of EBs was performed in ultra-low attachment plates (Corning, NY, USA) with differentiation induction medium (EB-medium) containing Knockout DMEM/F12, 20% KnockOut serum replacement, 2 mM GlutaMAX Supplement, 0.1 mM non-essential amino acids, 0.1 mM b-Mercaptoethanol (all from Gibco, Carlsbad, USA). For long-term culture of EBs for 15 days, EBs were transferred from ultra-low attachment plates to 0.1% gelatin-coated plates after day 7. Medium was changed every second day.

Immunostaining

Cells were fixed with 4% paraformaldehyde for 20 min, treated with PBS containing 1% BSA and 0.1% Triton X-100 (Bio-Rad, Munich, Germany) for 30 min, and then incubated overnight at 4°C with primary antibodies against OCT4 (clone C-10; Santa Cruz, Dallas, Texas, USA), GATA6 (clone D61E4; Cell Signaling, Danvers, USA), Brachyury (R&D Systems, Minneapolis, USA), and PAX6 (clone AD2.35; Santa Cruz, Dallas, USA). Secondary antibody staining was done at room temperature for 1 h with donkey anti-goat (Alexa Fluor 488), goat anti-rabbit (Alexa Fluor 594), and goat anti-mouse (Alexa Fluor 594), all from Invitrogen (Waltham, USA). Samples were counterstained with DAPI (10 ng/mL) for 15 min and imaged using an Axioplan 2 Fluorescence Microscope from Zeiss.

DNA methylation profiling

Genomic DNA was isolated with the NucleoSpin Tissue Kit (Macherey-Nagel, Düren, Germany) and quantified with a NanoDrop 2000 spectrophotometer (Thermo Fisher Scientific, Waltham, USA). 1.2 μg DNA was bisulfite converted and analyzed with Illumina EPIC BeadChip microarrays at Life & Brain (Bonn, Germany; dataset 1). Additionally, we used 114 DNA methylation profiles of iPSC and iPSC-derived cells (PSC, ENDO, MESO, ECTO, and EB) that were generated on Illumina HumanMethylation450 BeadChips by Progenitor Cell Biology Consortium (PCBC) of the National Heart, Lung and Blood Institute (dataset 2; Table S1) (Daily et al., 2017) from Gene Expression Omnibus (https://www.ncbi.nlm.nih.gov/geo; GSE85828). For comparison, we used DNAm profiles of iPSC-derived cells that were differentiated toward various cell types (dataset 3; Table S1). For the somatic cells, we used a selection of DNAm profiles that have been compiled for our previous work across many studies (dataset 4; Table S1) (Schmidt et al., 2020). Public DNAm profiles from cells undergoing reprogramming into iPSCs were downloaded from GEO (GSE54848; dataset 5; Table S1) (Ohnuki et al., 2014). Furthermore, we used DNAm profiles of iPSCs with HDC or LDC toward endoderm (GSE59091; dataset 6, for samples with replicas, we used always the mean values across all corresponding replicas) (Butcher et al., 2016).

The IDAT files of the Illumina BeadChips were loaded and pre-processed with minfi (Aryee et al., 2014) in R (4.1.3). Low-quality samples were removed (threshold: sum of the medians of the methylated and unmethylated channels <20), and the remaining samples were normalized with ssNoob (Triche et al., 2013). For samples where no IDAT files were available, we used already existing beta values or generated the beta values from the signal intensities. CpG sites on XY chromosomes, non-CG probes, and SNP-associated CpGs were not considered for further analysis. Furthermore, we only considered CpGs that were represented by the 450K and EPIC BeadChip platforms. The limma R package (3.48.0) was used for calculation of Benjamini-Hochberg adjusted p values and the MDS plots. Relevant DNAm changes were defined as showing at least 20% difference in mean beta values and an adjusted p value $\leq$ 0.05. Fisher exact test was performed with the R package GeneOverlap. The R packages ggplot2, ggrepel, ggbeeswarm, reshape2, ggExtra, ggsignif, cowplot, gprofiler2, ComplexHeatmap, and VennDiagram were used for graphical presentation.

Selection of epigenetic biomarkers

The selection of candidate marker CpGs is based on the R package CimpleG (https://github.com/CostaLab/CimpleG) (Maié et al., 2022). We selected CpG sites with high difference in mean beta values and low variances within the groups. The pluripotency score is based on the sum of DNAm at the three pluripotency-associated CpGs: cg00661673, cg00933813, and cg21699252. Since all these CpGs have lower DNAm in pluripotent cells, we calculated the complementary percentages for more intuitive application:

$$Pluripotency\ score = \left(1 - DNAm^{cg00661673}\right) + \left(1 - DNAm^{cg00933813}\right) + \left(1 - DNAm^{cg21699252}\right)$$

The deconvolution approach is based on non-negative matrix factorization, as described in our previous work (Frobel et al., 2018; Schmidt et al., 2020). As reference matrix we used either the mean DNAm values from the selection set or pyrosequencing data, respectively. We included Table S3, which allows the user to perform the reference-based deconvolution.

Transcriptomic analysis

RNA sequencing was performed by Life & Brain company (Bonn, Germany) using NovaSeq 6000 sequencer (100 bp/read). The FASTA files were checked with FastQC, and adapter sequences were trimmed using Trimmomatic. The alignment for the reads was done using STAR (hg38 genome build). Alternatively, count matrices were downloaded from the PCBC web portal (https://www.synapse.org/#!Synapse:syn2822494). Data were normalized with the variance-stabilizing transformation method from the DESeq2 package in R (Love et al., 2014). Differential gene expression analysis was performed with the same package using a Wald test (Benjamini-Hochberg adjusted p value < 0.05, absolute fold change >2). To correlate DNAm and gene expression data, we used the Illumina BeadChip annotation and merged the data by matching to Ensembl IDs, only considering CpGs in promoter regions (TSS1500 and TSS200).

To identify genes that are characteristic for the germ layers, we either used published marker sets for the three germ layer and stem cells (Maguire et al., 2013; Stronati et al., 2022) or we used a previously published single-cell RNA-seq dataset of human EBs (Han et al., 2018). All runs for day 8 EBs were merged, and counts were normalized. The Seurat package (v4) was used for quality control and filtration of cells with abnormal feature counts (Hao et al., 2021). Cells were clustered on K-nearest neighbor graph embedding using the Louvain algorithm, and representative markers of the individual clusters were identified using MAST (Finak et al., 2015). The identity of each cluster was annotated using Gene Ontology terms associated with marker genes using gprofiler2 (Kolberg et al., 2020), and clusters derived from the same germ layer were merged. Subsequently, differentially expressed genes having an adjusted p value < 0.05 and fold change >1.5 were considered as markers for the germ layers (Table S2).

For the PluriTest assay, we used the online PluriTest tool (https://www.pluritest.org/). RNA-seq FASTAQ files were uploaded to the website where they were pre-processed, aligned, and analyzed automatically using proprietary algorithm. The resulting pluripotency and novelty scores were plotted accordingly.

Pyrosequencing

Genomic DNA (500 ng) was bisulfite converted overnight using the EZ DNA Methylation Kit (Zymo) and eluted in 20 μL elution buffer. Primer (Metabion) was designed with the PyroMark Assay Design 2.0 Software (Qiagen; Table S4). Target sequences were amplified with the PyroMark PCR Kit (Qiagen) with 2.5 mM Mg^{2+} and a primer concentration of 0.3 μM. Pyrosequencing was performed on a Q96 ID pyrosequencer (Qiagen).

Semi-quantitative reverse-transcriptase PCR

Total RNA was isolated using the NucleoSpin RNA Plus Kit (Macherey-Nagel, Düren, Germany), quantified with a NanoDrop 2000 spectrophotometer (Thermo Fisher Scientific, Waltham, USA) and converted into cDNA using the High-Capacity cDNA Reverse Transcription Kit (Applied Biosystems, Waltham, USA). Semi-quantitative RT-PCR (qRT-PCR) was carried out using Power SYBR Green PCR Master Mix (Applied Biosystems, Waltham, USA) and gene-specific primers in a StepOnePlus machine (Applied Biosystems, Waltham, USA). Primers for *POU5F1* (OCT4*), GATA6, TBXT (*Brachyury*), PAX6*, and housekeeping gene *GAPDH* are provided in Table S5. ScoreCard analysis was performed with the TaqMan hPSC ScoreCard 96-well Kit (Thermo Fischer Scientific) according to the manufacturer's instructions.

Statistics

All statistical analysis was performed in R. For differential gene expression (DEseq2, Wald test) and methylation (limma, moderated t test) analysis, p values were adjusted using the Benjamini-Hochberg procedure. All adjusted p values smaller than 0.05 were considered as being significant. For comparison of pluripotency scores of LDC and HDC iPSCs, a Wilcoxon test was performed using the ggsignif R package.

SUPPLEMENTAL INFORMATION

Supplemental information can be found online at https://doi.org/10.1016/j.stemcr.2022.11.001.

AUTHOR CONTRIBUTIONS

M.S. performed analysis of DNAm profiles, established epigenetic signatures, and designed pyrosequencing assays. K.Z. and R.G. performed iPSC culture experiments and ScoreCard analysis. M.E.M. compiled training and validation datasets and performed bulk and single-cell gene expression analysis. W.W. designed and supervised the study. M.S., K.Z., M.E.M., and W.W. wrote the manuscript, and all authors approved the final version.

ACKNOWLEDGMENTS

This research was supported by the Federal Ministry of Education and Research (GO-Bio: Pluri-Screen, 16LW0017); the Deutsche Forschungsgemeinschaft (DFG, German Research Foundation 363055819/GRK2415; WA1706/11-1; WA1706/12-1 within CRU344; WA1706/14-1); by the START-Program of the Faculty of Medicine, RWTH Aachen (01/20); and the ForTra gGmbH für Forschungstransfer der Else Kröner-Fresenius-Stiftung.

CONFLICT OF INTERESTS

W.W. is cofounder of Cygenia GmbH that can provide service for various epigenetic signatures, including Epi-Pluri-Score analysis (www.cygenia.com). RWTH Aachen Medical School has claimed a patent application for GermLayerTracker and W.W., M.S., K.Z., and M.E.M. are listed as inventors.

Received: August 4, 2022
Revised: October 31, 2022
Accepted: November 1, 2022
Published: December 1, 2022

REFERENCES

Aryee, M.J., Jaffe, A.E., Corrada-Bravo, H., Ladd-Acosta, C., Feinberg, A.P., Hansen, K.D., and Irizarry, R.A. (2014). Minfi: a flexible and comprehensive Bioconductor package for the analysis of Infinium DNA methylation microarrays. Bioinformatics *30*, 1363–1369.

Bock, C., Kiskinis, E., Verstappen, G., Gu, H., Boulting, G., Smith, Z.D., Ziller, M., Croft, G.F., Amoroso, M.W., Oakley, D.H., et al. (2011). Reference Maps of human ES and iPS cell variation enable high-throughput characterization of pluripotent cell lines. Cell *144*, 439–452.

Bouma, M.J., van Iterson, M., Janssen, B., Mummery, C.L., Salvatori, D.C.F., and Freund, C. (2017). Differentiation-defective human induced pluripotent stem cells reveal strengths and limitations of the teratoma assay and in vitro pluripotency assays. Stem Cell Rep. *8*, 1340–1353.

Butcher, L.M., Ito, M., Brimpari, M., Morris, T.J., Soares, F.A.C., Ährlund-Richter, L., Carey, N., Vallier, L., Ferguson-Smith, A.C., and Beck, S. (2016). Non-CG DNA methylation is a biomarker for assessing endodermal differentiation capacity in pluripotent stem cells. Nat. Commun. *7*, 10458.

Cypris, O., Eipel, M., Franzen, J., Rösseler, C., Tharmapalan, V., Kuo, C.C., Vieri, M., Nikolić, M., Kirschner, M., Brümmendorf, T.H., et al. (2020). PRDM8 reveals aberrant DNA methylation in aging syndromes and is relevant for hematopoietic and neuronal differentiation. Clin. Epigenetics *12*, 125.

Daily, K., Ho Sui, S.J., Schriml, L.M., Dexheimer, P.J., Salomonis, N., Schroll, R., Bush, S., Keddache, M., Mayhew, C., Lotia, S., et al. (2017). Molecular, phenotypic, and sample-associated data to describe pluripotent stem cell lines and derivatives. Sci. Data *4*, 170030.

Dolgin, E. (2010). Putting stem cells to the test. Nat. Med. *16*, 1354–1357.

Elsafi Mabrouk, M.H., Goetzke, R., Abagnale, G., Yesilyurt, B., Salz, L., Cypris, O., Glück, P., Liesenfelder, S., Zeevaert, K., Ma, Z., et al. (2022). The spatial self-organization within pluripotent stem cell colonies is continued in detaching aggregates. Biomaterials *282*, 121389.

Finak, G., McDavid, A., Yajima, M., Deng, J., Gersuk, V., Shalek, A.K., Slichter, C.K., Miller, H.W., McElrath, M.J., Prlic, M., et al. (2015). MAST: a flexible statistical framework for assessing transcriptional changes and characterizing heterogeneity in single-cell RNA sequencing data. Genome Biol. *16*, 278.

Franzen, J., Georgomanolis, T., Selich, A., Kuo, C.-C., Stöger, R., Brant, L., Mulabdić, M.S., Fernandez-Rebollo, E., Grezella, C., Ostrowska, A., et al. (2021). DNA methylation changes during long-term in vitro cell culture are caused by epigenetic drift. Commun. Biol. *4*, 598.

Frobel, J., Božić, T., Lenz, M., Uciechowski, P., Han, Y., Herwartz, R., Strathmann, K., Isfort, S., Panse, J., Esser, A., et al. (2018). Leukocyte counts based on DNA methylation at individual cytosines. Clin. Chem. *64*, 566–575.

Gifford, C.A., Ziller, M.J., Gu, H., Trapnell, C., Donaghey, J., Tsankov, A., Shalek, A.K., Kelley, D.R., Shishkin, A.A., Issner, R., et al. (2013). Transcriptional and epigenetic dynamics during specification of human embryonic stem cells. Cell *153*, 1149–1163.

Goetzke, R., Franzen, J., Ostrowska, A., Vogt, M., Blaeser, A., Klein, G., Rath, B., Fischer, H., Zenke, M., and Wagner, W. (2018). Does soft really matter? Differentiation of induced pluripotent stem cells into mesenchymal stromal cells is not influenced by soft hydrogels. Biomaterials *156*, 147–158.

Han, X., Chen, H., Huang, D., Chen, H., Fei, L., Cheng, C., Huang, H., Yuan, G.C., and Guo, G. (2018). Mapping human pluripotent stem cell differentiation pathways using high throughput single-cell RNA-sequencing. Genome Biol. *19*, 47.

Han, Y., Franzen, J., Stiehl, T., Gobs, M., Kuo, C.-C., Nikolić, M., Hapala, J., Koop, B.E., Strathmann, K., Ritz-Timme, S., et al. (2020). New targeted approaches for epigenetic age predictions. BMC Biol. *18*, 71.

Hao, Y., Hao, S., Andersen-Nissen, E., Mauck, W.M., 3rd, Zheng, S., Butler, A., Lee, M.J., Wilk, A.J., Darby, C., Zager, M., et al. (2021). Integrated analysis of multimodal single-cell data. Cell *184*, 3573–3587.e29.

Houseman, E.A., Accomando, W.P., Koestler, D.C., Christensen, B.C., Marsit, C.J., Nelson, H.H., Wiencke, J.K., and Kelsey, K.T. (2012). DNA methylation arrays as surrogate measures of cell mixture distribution. BMC Bioinf. *13*, 86.

International Stem Cell Initiative (2018). Assessment of established techniques to determine developmental and malignant potential of human pluripotent stem cells. Nat. Commun. *9*, 1925.

Kolberg, L., Raudvere, U., Kuzmin, I., Vilo, J., and Peterson, H. (2020). gprofiler2 – an R package for gene list functional enrichment analysis and namespace conversion toolset g:Profiler. F1000Res. *9*, ELIXIR-709.

Lenz, M., Goetzke, R., Schenk, A., Schubert, C., Veeck, J., Hemeda, H., Koschmieder, S., Zenke, M., Schuppert, A., and Wagner, W. (2015). Epigenetic biomarker to support classification into pluripotent and non-pluripotent cells. Sci. Rep. *5*, 8973.

Love, M.I., Huber, W., and Anders, S. (2014). Moderated estimation of fold change and dispersion for RNA-seq data with DESeq2. Genome Biol. *15*, 550.

Maguire, C.T., Demarest, B.L., Hill, J.T., Palmer, J.D., Brothman, A.R., Yost, H.J., and Condic, M.L. (2013). Genome-wide analysis reveals the unique stem cell identity of human amniocytes. PLoS One *8*, e53372.

Maié, T., Schmidt, M., Erz, M., Wagner, W., and Costa, I.G. (2022). CimpleG: finding simple CpG methylation signatures. Preprint at bioRxiv. https://doi.org/10.1101/2022.09.12.507513.

Moss, J., Magenheim, J., Neiman, D., Zemmour, H., Loyfer, N., Korach, A., Samet, Y., Maoz, M., Druid, H., Arner, P., et al. (2018). Comprehensive human cell-type methylation atlas reveals origins of circulating cell-free DNA in health and disease. Nat. Commun. *9*, 5068.

Muller, F.J., Goldmann, J., Löser, P., and Loring, J.F. (2010). A call to standardize teratoma assays used to define human pluripotent cell lines. Cell Stem Cell *6*, 412–414.

Muller, F.J., Schuldt, B.M., Williams, R., Mason, D., Altun, G., Papapetrou, E.P., Danner, S., Goldmann, J.E., Herbst, A., Schmidt, N.O., et al. (2011). A bioinformatic assay for pluripotency in human cells. Nat. Methods *8*, 315–317.

Ng, E.S., Davis, R.P., Azzola, L., Stanley, E.G., and Elefanty, A.G. (2005). Forced aggregation of defined numbers of human embryonic stem cells into embryoid bodies fosters robust, reproducible hematopoietic differentiation. Blood *106*, 1601–1603.

O'Shea, O., Steeg, R., Chapman, C., Mackintosh, P., and Stacey, G.N. (2020). Development and implementation of large-scale quality control for the European bank for induced Pluripotent Stem Cells. Stem Cell Res. *45*, 101773.

Ohnuki, M., Tanabe, K., Sutou, K., Teramoto, I., Sawamura, Y., Narita, M., Nakamura, M., Tokunaga, Y., Nakamura, M., Watanabe, A., et al. (2014). Dynamic regulation of human endogenous retroviruses mediates factor-induced reprogramming and differentiation potential. Proc. Natl. Acad. Sci. USA *111*, 12426–12431.

Roadmap Epigenomics Consortium, Kundaje, A., Meuleman, W., Ernst, J., Bilenky, M., Yen, A., Heravi-Moussavi, A., Kheradpour, P., Zhang, Z., Wang, J., et al. (2015). Integrative analysis of 111 reference human epigenomes. Nature *518*, 317–330.

Salomonis, N., Dexheimer, P.J., Omberg, L., Schroll, R., Bush, S., Huo, J., Schriml, L., Ho Sui, S., Keddache, M., Mayhew, C., et al. (2016). Integrated genomic analysis of diverse induced pluripotent stem cells from the progenitor cell Biology Consortium. Stem Cell Rep. *7*, 110–125.

Schmidt, M., Maié, T., Dahl, E., Costa, I.G., and Wagner, W. (2020). Deconvolution of cellular subsets in human tissue based on targeted DNA methylation analysis at individual CpG sites. BMC Biol. *18*, 178.

Sontag, S., Bocova, L., Hubens, W.H.G., Nüchtern, S., Schnitker, M., Look, T., Schröder, K.M., Plümäkers, B., Tharmapalan, V., Wessiepe, M., et al. (2022). Toward clinical application of leukocyte counts based on targeted DNA methylation analysis. Clin. Chem. *68*, 646–656.

Steeg, R., Mueller, S.C., Mah, N., Holst, B., Cabrera-Socorro, A., Stacey, G.N., De Sousa, P.A., Courtney, A., and Zimmermann, H. (2021). EBiSC best practice: how to ensure optimal generation, qualification, and distribution of iPSC lines. Stem Cell Rep. *16*, 1853–1867.

Stronati, E., Giraldez, S., Huang, L., Abraham, E., McGuire, G.R., Hsu, H.T., Jones, K.A., and Estarás, C. (2022). YAP1 regulates the self-organized fate patterning of hESC-derived gastruloids. Stem Cell Rep. *17*, 211–220.

Triche, T.J., Jr., Weisenberger, D.J., Van Den Berg, D., Laird, P.W., and Siegmund, K.D. (2013). Low-level processing of Illumina Infinium DNA methylation BeadArrays. Nucleic Acids Res. *41*, e90.

Tsankov, A.M., Akopian, V., Pop, R., Chetty, S., Gifford, C.A., Daheron, L., Tsankova, N.M., and Meissner, A. (2015). A qPCR ScoreCard quantifies the differentiation potential of human pluripotent stem cells. Nat. Biotechnol. *33*, 1182–1192.

Wagner, W. (2022). How to translate DNA methylation biomarkers into clinical practice. Front. Cell Dev. Biol. *10*, 854797.

Willmann, C.A., Hemeda, H., Pieper, L.A., Lenz, M., Qin, J., Joussen, S., Sontag, S., Wanek, P., Denecke, B., Schüler, H.M., et al. (2013). To clone or not to clone? Induced pluripotent stem cells can be generated in bulk culture. PLoS One *8*, e65324.

Zeevaert, K., Goetzke, R., Elsafi Mabrouk, M.H., Schmidt, M., Maaßen, C., Henneke, A.-C., He, C., Gillner, A., Zenke, M., and Wagner, W. (2022). YAP1 is essential for self-organized differentiation of pluripotent stem cells. Preprint at bioRxiv. https://doi.org/10.1101/2022.09.29.510043.

Stem Cell Reports, Volume *18*

Supplemental Information

Epigenetic biomarkers to track differentiation of pluripotent stem cells

Marco Schmidt, Kira Zeevaert, Mohamed H. Elsafi Mabrouk, Roman Goetzke, and Wolfgang Wagner

Supplemental Figures

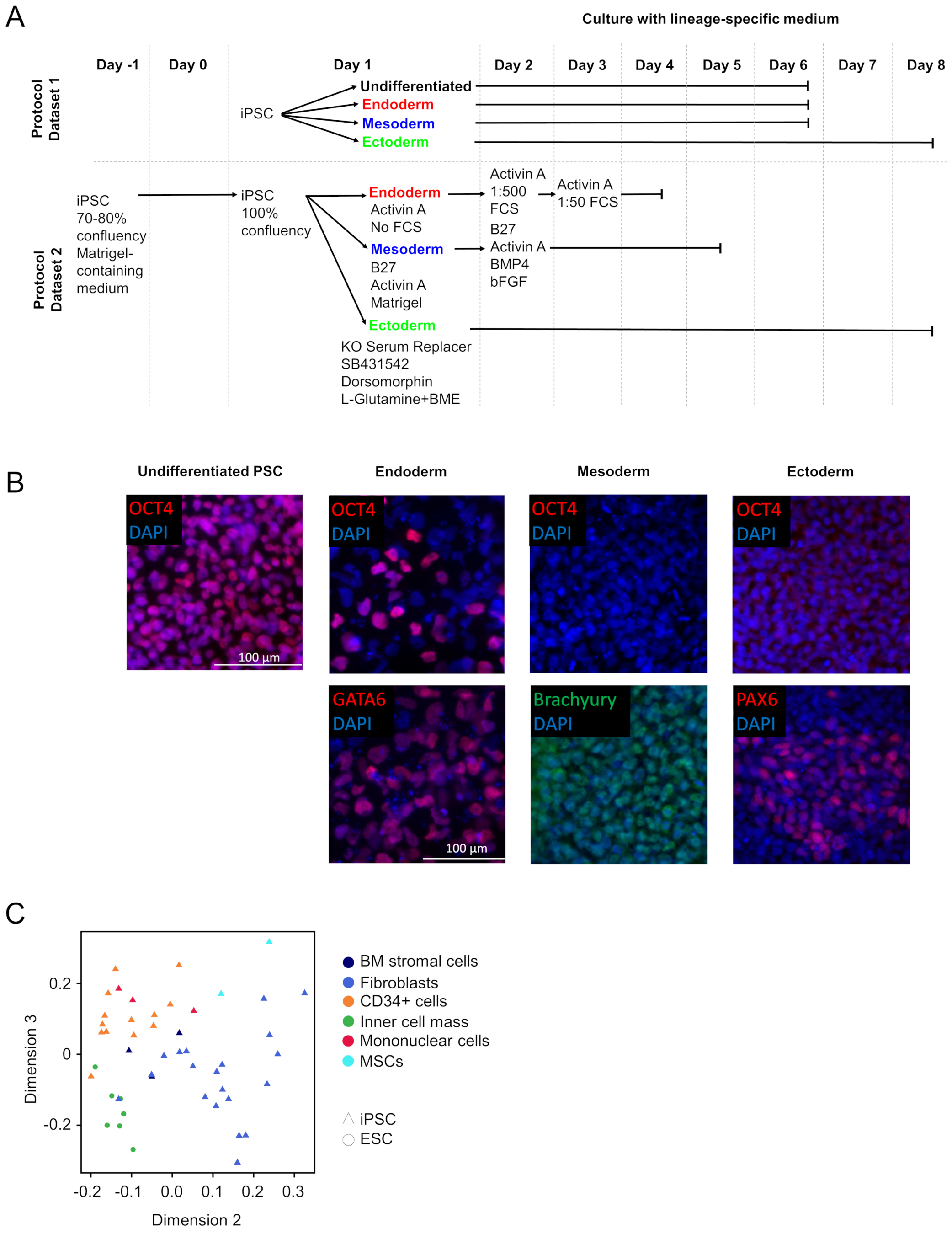

Figure S1: Trilineage differentiation of pluripotent stem cells (related to Figure 1)

(A) Trilineage differentiation protocols that were used for own iPSCs (dataset 1; STEMdiff Trilineage Differentiation Kit; Stemcell Technologies) and in the public dataset (dataset 2; GSE85828) (Daily et al., 2017).

(B) Immunofluorescence staining of exemplary differentiated cells (protocol dataset 1) stained with antibodies against OCT4 (stem cells), GATA6 (endoderm), Brachyury (mesoderm), and PAX6 (ectoderm). Nuclear staining with DAPI.

(C) Multidimensional scaling (MDS) plot of the top 10,000 most variable CpGs from own (dataset 1) and public DNAm profiles (dataset 2) (Daily et al., 2017) of undifferentiated stem cells only. Cell lines seem to cluster based on the cells used for reprogramming. SC12-040 was removed as being a prominent outlier.

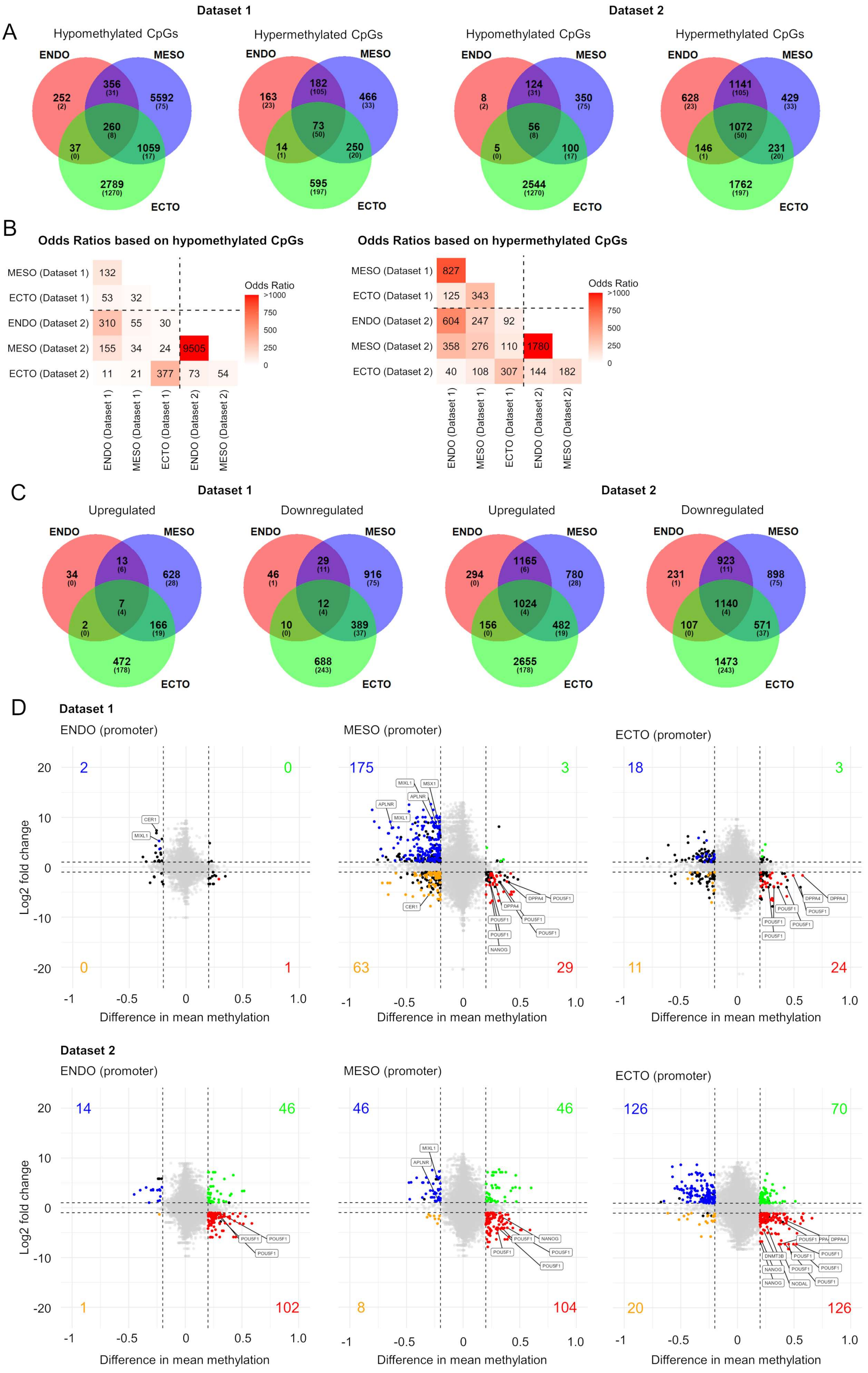

Figure S2 continues on the next page.

E

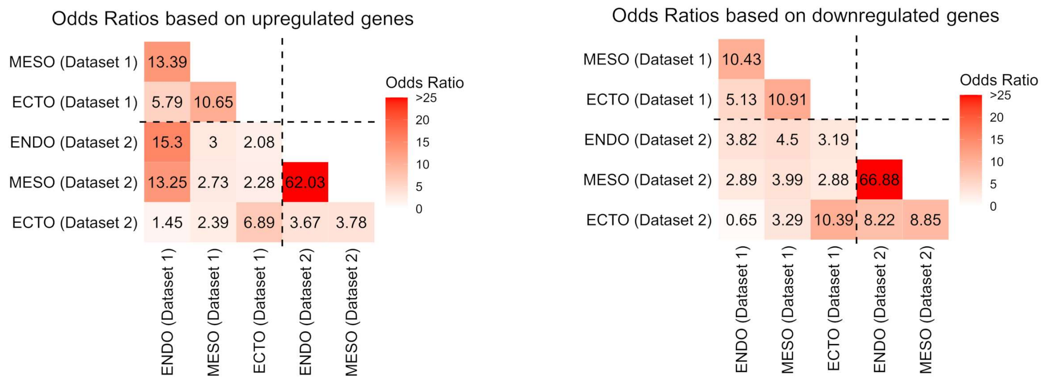

Figure S2: Overlap of DNA methylation changes in different datasets (related to Figure 1)

(A) Venn diagrams depict the number of significant hypo- and hypermethylated CpGs that are overlapping during differentiation towards endoderm (ENDO), mesoderm (MESO), and ectoderm (ECTO). This analysis was performed for dataset 1 and dataset 2, in parallel. Additionally, the overlap between the datasets is indicated in parenthesis.

(B) To estimate the relationship between the DNAm profiles of the differentiated samples we performed pairwise comparison of differentially methylated probes in datasets 1 and dataset 2 (for hypo- and hypermethylated sites, separately). The odds ratio of Fisher-Exact-Test provide the likelihood of getting the shared differentially methylated CpGs, given the total sample size (all CpGs). The high odds ratio between ENDO and MESO from dataset 2 indicates that the DNA methylation changes in these samples are highly overlapping.

(C) Venn diagrams provide the number of significant gene expression changes during differentiation into endoderm, mesoderm, and ectoderm. This analysis was performed for dataset 1 and dataset 2, separately. Additionally, the overlap between the datasets is indicated in parenthesis.

(D) Integrative analysis of DNA methylation and gene expression changes. Only CpGs in promoter regions (TSS1500, TSS200) are considered. Each dot represents a gene-CpG-pair (genes as well as CpGs might be duplicated). Colored dots depict pairs with a significant difference in DNAm and gene expression changes during differentiation.

(E) Heatmap of pair-wise odds ratio between differentially expression genes across differentiation modalities (Fisher-Exact Test). In dataset 2 the gene expression changes are significantly overlapping during endodermal and mesodermal differentiation.

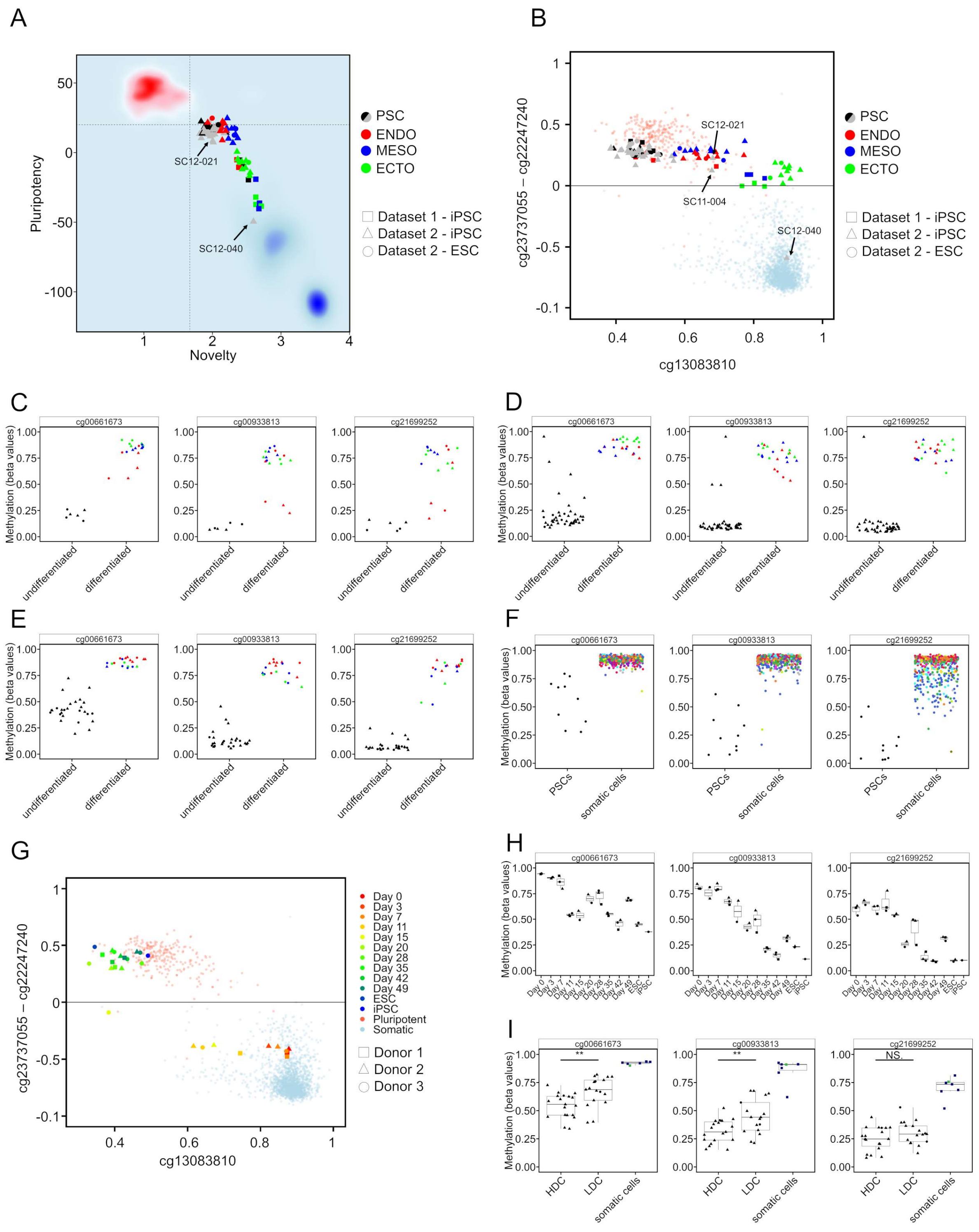

Figure S3, legend on the next page.

Figure S3: Comparison of different signatures for pluripotency (related to Figure 2)

(A) PluriTest analysis was performed with the online PluriTest tool (https://www.pluritest.org/). RNA-seq FASTAQ files were uploaded to the website for pre-processing, alignment, and automated analysis using the proprietary algorithm. The results showed that most stem cells, mesoderm, and endoderm samples had a similar pluripotency score. PluriTest was not able to distinguish between them and the predictions did not cluster with pluripotent cloud of the empirical density map. Furthermore, two of three iPSCs lines from dataset 1 and the outlier cell line SC12-040 had scores were indicated as failed.

(B) Epi-Pluri-Score analysis is based on DNAm at three specific CpGs. One of these CpGs was localized within the pluripotency-associated gene *POU5F1* (also known as *OCT4*). Furthermore, the difference in DNAm levels of CpGs in *ANKRD46* and *C14orf115* was determined and combined as Epi-Pluri-Score (Lenz et al., 2015). The dots in the background refer to DNAm profiles (all Illumina HumanMethylation27 BeadChip platform) of 264 pluripotent and 1,951 non-pluripotent cell preparations, respectively (Lenz et al., 2015). Notably, Epi-Pluri-Score classified all cell preparations as pluripotent, while early differentiation events can be tracked by increasing DNAm in *POU5F1.* For the three iPSC samples that were previously identified as outliers the sample IDs are again highlighted (GSM2285159, dataset 2). Particularly the sample SC12-040 was clearly classified as non-pluripotent, and this sample clustered also apart in the MDS and PCA plots (Figure 1A,C) and apparently also revealed an aberrant karyotype (Salomonis et al., 2016).

(C) DNA methylation levels for the undifferentiated and differentiated cells of the selection set for the three CpGs that were selected for the pluripotency score. Each of the CpGs could discern pluripotent and non-pluripotent cells.

(D) DNAm levels at the three candidate CpGs for the pluripotency score of the remaining samples from dataset 2 (Daily et al., 2017).

(E) DNAm levels at the three candidate CpGs for the pluripotency score in various iPSC-derived cell types (dataset 3; Table S1).

(F) DNAm levels at the three candidate CpGs for the pluripotency score for a collection of various somatic cell types (dataset 4; Table S1) (Schmidt et al., 2020).

(G) Epi-Pluri-Score analysis. Depicted are the same samples from Figure 2F and S3G. In contrast to the pluripotency score, the Epi-Pluri-Score changes abruptly between day 15 and 20.

(H) DNAm levels at the three candidate CpGs for the pluripotency score for a collection of samples during reprogramming of fibroblasts into iPSCs (dataset 5; Table S1) (Ohnuki et al., 2014).

(I) DNAm levels at the three candidate CpGs for the pluripotency score for iPSC samples (dataset 6; Table S1) (Butcher et al., 2016), which have been grouped into high differentiation capacity (HDC) and low differentiation capacity (LDC) toward endoderm. The primary donor samples (fibroblasts and endothelial precursors) are shown for comparison. P-values were calculated with Wilcoxon-Test (** $p < 0.01$).

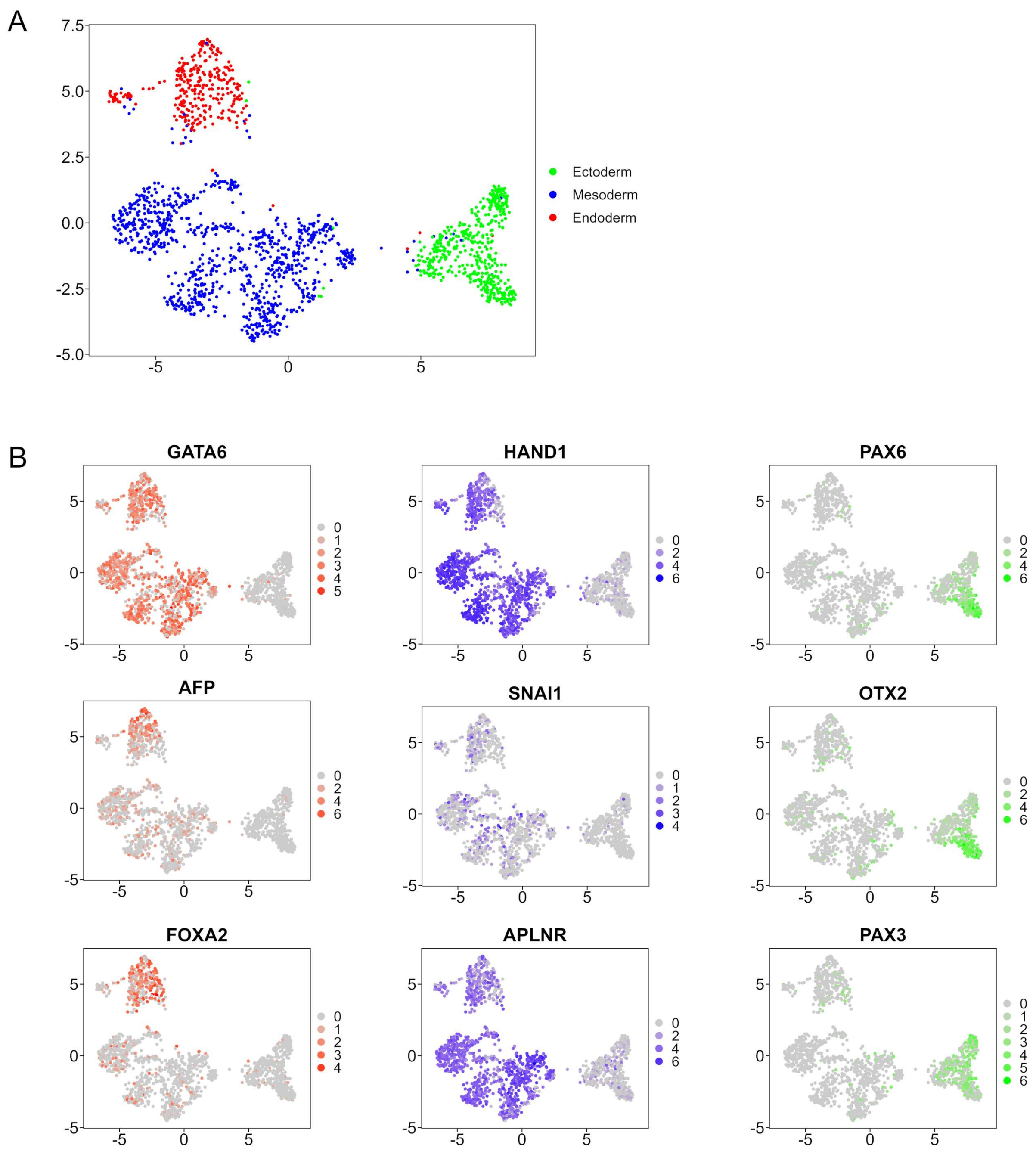

Figure S4: Identification of gene signatures for germ layers in EBs (related to Figure 4)

(A) To identify marker genes for endodermal, mesodermal, and ectodermal differentiation in spontaneous differentiation, we used single-cell RNA sequencing data of EBs at day eight of spontaneous differentiation (Han et al., 2018). Uniform Manifold Approximation and Projection (UMAP) representation of the data demonstrated that the cells could be classified in three distinct clusters.

(B) UMAP representation of normalized expression levels for endoderm markers (*GATA6*, *AFP*, *FOXA2*), mesoderm markers (*HAND1*, *SNAI1*, *APLNR*), and ectoderm markers (*PAX6*, *OTX2*, *PAX3*) demonstrates that these clusters were indeed associated with the respective germ layers. The same results were also observed for gene ontology analysis (not presented). For each of these clusters we subsequently selected genes that are significantly higher expressed than in the other clusters: for endoderm 279 genes, mesoderm 425 genes, and ectoderm 516 genes (Table S2).

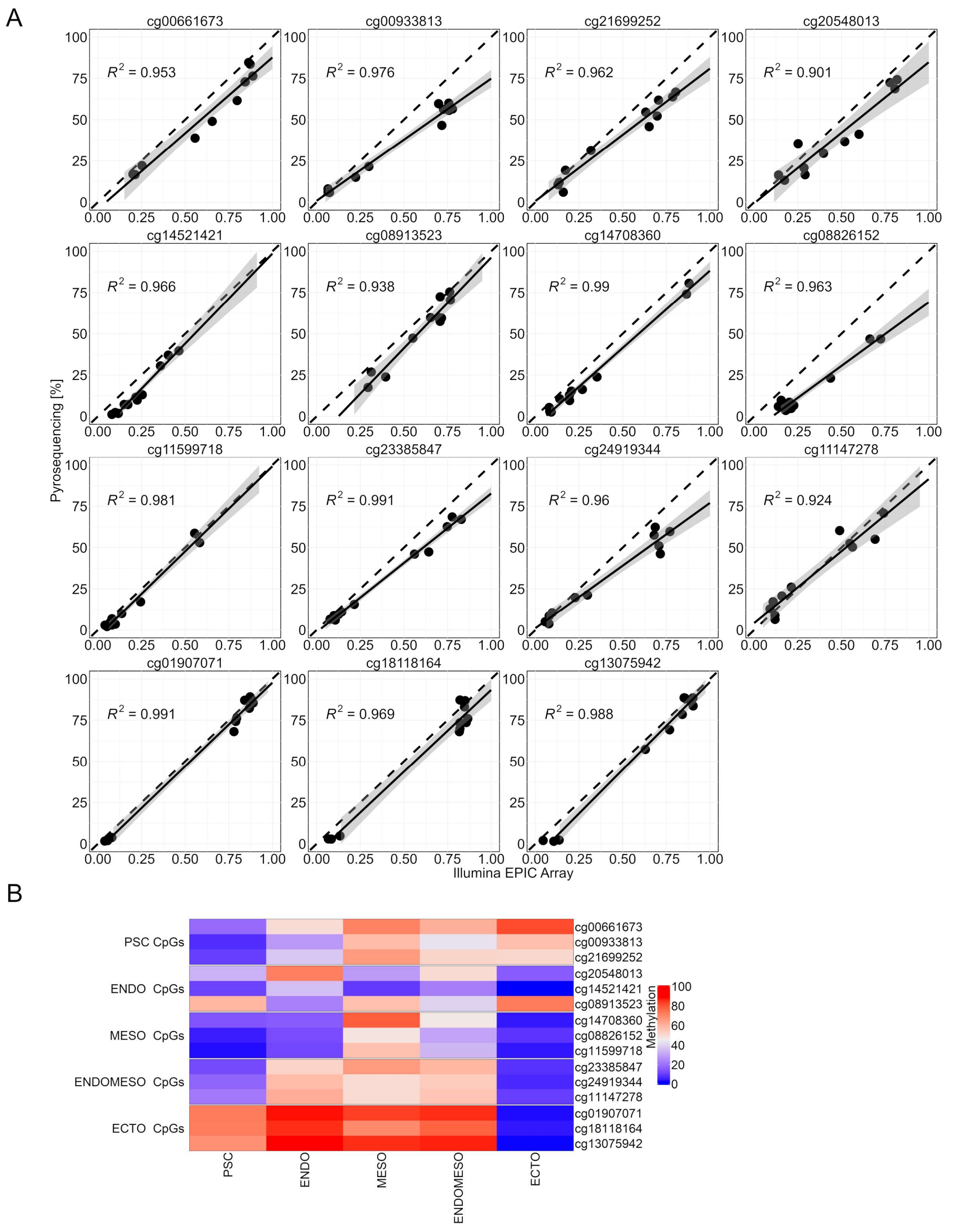

Figure S5: Pyrosequencing assays for GermLayerTracker CpGs (related to Figure 5)

(A) Comparison of DNAm values measured with pyrosequencing and the Illumina EPIC BeadChip technology.

(B) Reference values based on pyrosequencing for deconvolution of germ layers. Depicted are mean values of the three cell lines from the directed 2D differentiation.

Table S1: List of all datasets used for selection and validation of the DNA methylation signatures

This table is provided as separate Excel sheet.

Table S2: List of germ layer signature genes derived from publically available sc-RNA-seq data for embryoid bodies, related to Figure 4

This table is provided as separate Excel sheet.

Table S3: Excel sheet for deconvolution of germ layer composition

This table is provided as separate Excel sheet.

Table S4: Pyrosequencing primer, related to Figure 5.

NAME	DNA SEQUENCE
cgSC1 For	GGTTGGAGTGTATTGGTGTAA
cgSC1 Rev	Biotin-AATCCCAACCTTTATACATATTAATTCTT
cgSC1 Seq	GTTGAGATTATAGGTGTGA
cgSC2 For	AGGTTGGTTATGAATTTTTGGTTTTAAGTA
cgSC2 Rev	Biotin- ATACCCTACCTTCCTTTCATTTATATTC
cgSC2 Seq	TTGGGATTATAGGTGTG
cgSC3 For	GATGTTGAGGGTTAGGGGGTAATT
cgSC3 Rev	Biotin- CCTAAAACTCTAAAAATCTTTCTCCCTAAA
cgSC3 Seq	TGAAGGTTTTTTTAGTTTTGA
cgE1 For	GAATAGTATATGGTTGGTTGGGAAAGT
cgE1 Rev	Biotin- CCAAAAAAAAAAAATACCTTTACTATCACT
cgE1 Seq	AGGAGTTATTTTATTATATTGGAG
cgE2 For	GGGATGTTGTGGATGGTAAAA
cgE2 Rev	Biotin- ACTCCCACATCTAAACACCTAA
cgE2 Seq	AGGGGTGTGGGAAGT
cgE3 For	GGGAGAGGGATTTATTATTAGGT
cgE3 Rev	Biotin- ACCCCCTCCTTCAACTATAAT
cgE3 Seq	GGTTTGAGAAAGAAGTTAG
cgM1 For	AGGGTAAGGTTGTTTTGTTTAGTTTAT
cgM1 Rev	Biotin- TCATACCTTTAAAACCCACAACTAAAAT
cgM1 Seq	ATTAGGGTTTTGGTTTTATT
cgM2 For	TGAGTTTGGTTAGTTTAGTTATAGGT
cgM2 Rev	Biotin- CATCCCTAAAACAAACAAAAAACAATT
cgM2 Seq	ATTTGTTGTTGAGGTTTTTAATA
cgM3 For	ATGGTTTGGTATAGAAAGTTTATGG
cgM3 Rev	Biotin- ATACTTTCATCTCTTCTAATACCTTTAAC
cgM3 Seq	GTTTTGTGGGTGGGG
cgEM1 For	GAATAAGATATGGTTTTTGGATTTGAGTA
cgEM1 Rev	Biotin- AAATTTTCCTCTCCTACATCTCTCA
cgEM1 Seq	GTGTTATAAGGTTTTGTTAGTT
cgEM2 For	Biotin- AGTTTTTTGATTATAAAAGGTATAGAGTGT
cgEM2 Rev	ACTCAAAAAAATCACCATAAATCACTATC
cgEM2 Seq	ACAACTAAACTTCTTTATCATATAT
cgEM3_C For	TGTTAGTAAATGGGGAAGATATAAAAGTT

cgEM3_C Rev	Biotin- AATTCCTACCCAACTCAAACTCATCTA
cgEM3_C Seq	GAGTTGATTTTGAAAGGT
cgEC1 For	GGGGTTTTGAAAGTAAATGTGT
cgEC1 Rev	Biotin- TTCCAACTCACTAAAAAACACTTC
cgEC1 Seq	AGTAAATGTGTTGAAAGTT
cgEC2 For	AGTGGGAGTAAATGAGTTTAGT
cgEC2 Rev	Biotin- CAATTTCAAAATCTCCATCTCAAAATATCA
cgEC2 Seq	TTTTAGGGTAAGAAAATATAGATAG
cgEC3 For	GGGAGATTTTAGTTTTTTTTGTAGGG
cgEC3 Rev	Biotin- CCCAATATTATAATTCTTAACACCTCTCAT
cgEC3 Seq	AGTTTTTTTTGTAGGGATTTT

Table S5: RT-qPCR primer, related to Figure 5.

NAME	DNA SEQUENCE
POU5F1 For	GGGGGTTCTATTTGGGAAGGTA
POU5F1 Rev	ACCCACTTCTGCAGCAAGGG
GATA6 For	CTCAGTTCCTACGCTTCGCAT
GATA6 Rev	GTCGAGGTCAGTGAACAGCA
TBXT For	CAGTGGCAGTCTCAGGTTAAGAAGGA
TBXT Rev	CGCTACTGCAGGTGTGAGCAA
PAX6 For	TCGAAGGGCCAAATGGAGAAGAGAAG
PAX6 Rev	GGTGGGTTGTGGAATTGGTTGGTAGA
GAPDH For	GAAGGTGAAGGTCGGAGTC
GAPDH Rev	GAAGATGGTGATGGGATTTC

Supplemental References

Daily, K., Ho Sui, S.J., Schriml, L.M., Dexheimer, P.J., Salomonis, N., Schroll, R., Bush, S., Keddache, M., Mayhew, C., Lotia, S., *et al.* (2017). Molecular, phenotypic, and sample-associated data to describe pluripotent stem cell lines and derivatives. Sci Data *4*, 170030.

Han, X., Chen, H., Huang, D., Chen, H., Fei, L., Cheng, C., Huang, H., Yuan, G.C., and Guo, G. (2018). Mapping human pluripotent stem cell differentiation pathways using high throughput single-cell RNA-sequencing. Genome Biol *19*, 47.

Lenz, M., Goetzke, R., Schenk, A., Schubert, C., Veeck, J., Hemeda, H., Koschmieder, S., Zenke, M., Schppert, A., and Wagner, W. (2015). Epigenetic biomarker to support classification into pluripotent and non-pluripotent cells. Scientific Reports *5*, 8973.

Ohnuki, M., Tanabe, K., Sutou, K., Teramoto, I., Sawamura, Y., Narita, M., Nakamura, M., Tokunaga, Y., Nakamura, M., Watanabe, A., *et al.* (2014). Dynamic regulation of human endogenous retroviruses mediates factor-induced reprogramming and differentiation potential. Proc Natl Acad Sci U S A *111*, 12426-12431.

Salomonis, N., Dexheimer, P.J., Omberg, L., Schroll, R., Bush, S., Huo, J., Schriml, L., Ho Sui, S., Keddache, M., Mayhew, C., *et al.* (2016). Integrated Genomic Analysis of Diverse Induced Pluripotent Stem Cells from the Progenitor Cell Biology Consortium. Stem Cell Reports *7*, 110-125.
